# Egg Case Silk Gene Sequences from *Argiope* Spiders: Evidence for Multiple Loci and a Loss of Function Between Paralogs

**DOI:** 10.1534/g3.117.300283

**Published:** 2017-11-10

**Authors:** R. Crystal Chaw, Matthew Collin, Marjorie Wimmer, Kara-Leigh Helmrick, Cheryl Y. Hayashi

**Affiliations:** * Department of Biology, University of California, Riverside, California 92521; † Division of Invertebrate Zoology, American Museum of Natural History, New York, New York 10024; ‡ Sackler Institute for Comparative Genomics, American Museum of Natural History, New York, New York 10024

**Keywords:** evolution, gene family, spider silk, tubuliform spidroin, TuSp1

## Abstract

Spiders swath their eggs with silk to protect developing embryos and hatchlings. Egg case silks, like other fibrous spider silks, are primarily composed of proteins called spidroins (spidroin = spider-fibroin). Silks, and thus spidroins, are important throughout the lives of spiders, yet the evolution of spidroin genes has been relatively understudied. Spidroin genes are notoriously difficult to sequence because they are typically very long (≥ 10 kb of coding sequence) and highly repetitive. Here, we investigate the evolution of spider silk genes through long-read sequencing of Bacterial Artificial Chromosome (BAC) clones. We demonstrate that the silver garden spider *Argiope argentata* has multiple egg case spidroin loci with a loss of function at one locus. We also use degenerate PCR primers to search the genomic DNA of congeneric species and find evidence for multiple egg case spidroin loci in other *Argiope* spiders. Comparative analyses show that these multiple loci are more similar at the nucleotide level within a species than between species. This pattern is consistent with concerted evolution homogenizing gene copies within a genome. More complicated explanations include convergent evolution or recent independent gene duplications within each species.

Throughout their lifetimes, spiders rely on silks for a variety of crucial tasks. One such task is wrapping egg clutches with silk to protect developing embryos and subsequent hatchlings from biotic and abiotic threats (*e.g.*, Austin 1985). Most species produce egg sac wrappings in a specialized set of silk glands that are called tubuliform glands (also known as cylindrical glands). One of the most highly expressed genes in tubuliform glands is the gene that encodes the structural protein tubuliform spidroin, or TuSp (synonymous with CySp, Cylindrical Spidroin; Garb and Hayashi 2005, Zhao *et al.* 2006, Casem *et al.* 2010, Chaw *et al.* 2016).

Spidroins are a family of spider-specific fibroins. Insights into the function and evolution of spidroin genes are mostly based on cDNAs (*e.g.*, Gatesy *et al.* 2001, Rising *et al.* 2007, Starrett *et al.* 2012), which lack information on the surrounding genomic regions and often represent truncated, partial-length transcripts that are a small fraction of the total length of the gene. Tubuliform spidroin genes are one of the few exceptions, with two annotated full-length transcripts from *Argiope bruennichi* [Zhao *et al.* 2006; but see Rising *et al.* (2006) and Han and Nakagaki (2013)].

The full-length *TuSp* transcripts from *A. bruennichi* and the truncated *TuSp1* cDNAs from various species indicate that *TuSp* protein-coding sequence has a modular organization that is similar to other spidroins. *TuSp* is dominated by a central repetitive region that is flanked by much shorter, nonrepetitive amino (N)- and carboxyl (C)-terminal-encoding regions (Garb and Hayashi 2005; Hu *et al.* 2005; Tian and Lewis 2005; Huang *et al.* 2006; Zhao *et al.* 2006). The tandem-arrayed repeats of *TuSp1* repetitive regions show extensive intragenic homogenization, making *TuSp1* repeats easily distinguishable by species (Garb and Hayashi 2005).

Studies of the full-length *TuSp* transcripts have not been definitive in diagnosing alleles and gene copies of tubuliform spidroin genes. By contrast, multiple loci have been documented for major ampullate spidroin 1, minor ampullate spidroin, and aciniform spidroin genes through genomic DNA studies (Ayoub and Hayashi 2008; Gaines and Marcotte 2008; Chaw *et al.* 2014; Vienneau-Hathaway *et al.* 2017). To better understand spidroin domain architecture and homogenization within and between gene copies, we characterized complete *TuSp1* loci from *A. argentata*. The repeat sequences within a *TuSp1* gene are nearly identical, consistent with previous studies showing intragenic concerted evolution (Garb and Hayashi 2005). We also demonstrate that there are multiple *TuSp1* loci in genomic DNAs of *A. argentata* and congeneric spiders.

## Materials and Methods

### Genomic DNA library construction, pool screening, and positive clone identification

Bacterial Artificial Chromosome (BAC) library construction was described in Chaw *et al.* (2017). Briefly, intact nuclei were purified from a previously snap-frozen mature virgin *A. argentata* spider. Plug preparation, DNA size selection, and cloning were performed following the general methods of Luo and Wing (2003). BAC transformations were combined, titered to ∼1500–2000 colonies, and grown on agar plates. Colonies were scraped, pooled together, and split between a glycerol archive and a sample for DNA extraction. Total BAC DNA extractions followed standard alkaline lysis conditions (Sambrook *et al.* 1989).

BAC pool DNAs were screened using standard PCR conditions with Amplitaq gold Taq polymerase (Thermo Fisher Scientific). Screening primers were from Garb and Hayashi (2005) (Supplemental Material, Table S1) and were designed to the repetitive region of an *A. argentata TuSp1* cDNA (GenBank accession AY953071). Two PCR-positive BAC pools were robotically arrayed into 48 (24 per pool) 384-well plates by plating a dilution of the corresponding glycerol archive on 22.5 × 22.5 cm semisolid media plates, and incubating at 37° for 18 hr (Genetix Q-bot; Molecular Devices). All 48 plates were subsequently gridded onto nitrocellulose filter membrane for DNA hybridization. Single BAC clones of interest were identified with ^32^P-radiolableled *A. argentata TuSp1* PCR amplicons, following the methods described by Fang *et al.* (2010).

### BAC sequencing and assembly

BAC sequencing was described in Chaw *et al.* (2017). Briefly, DNA was extracted from single BAC clones that were positive for *TuSp1*, and the extracted DNAs were pooled in equimolar amounts (Saski *et al.* 2015). The DNA pools were sequenced on the Pacific Biosciences RSII+ single molecule sequencer with P6-C4 chemistry. Raw sequence data were filtered to remove short reads (< 1000 bp) and *de novo* assembled with CANU, using the self-correction module and default settings (Koren *et al.* 2016). Manual editing and consensus preparations were performed with Consed (Gordon *et al.* 1998).

In the assembly results, three contigs were recovered from three different BAC clones. The first contig contained a locus of *TuSp1* and had five frame shifts that were attributed to sequencing artifacts in the ∼9.5 kb coding region. The frame shifts all occurred in the repetitive region, which has tandem repeats that are highly homogenized with each other (Garb and Hayashi 2005). Thus, the repeat units were aligned, and the frame shifts were resolved (two nucleotide additions and three deletions) according to the majority-rule consensus repeat.

The other two contigs were identical, with one nested within the other. These contigs were collapsed into a single contig that contained an unexpectedly short (< 1.5 kb) second *TuSp1* locus that did not code through. The accuracy of the assembled contig was verified by separate PCR amplification and direct sequencing of genomic DNAs extracted from two individual *A. argentata* (see below for extraction protocol). Primers were designed to amplify the entire second locus (Table S1).

### Comparative analyses of TuSp termini

We searched for *TuSp* (synonymous with *CySp*) termini-encoding regions in public databases with a text search of gene names and a tBLASTx search. The nucleotide sequences of *A. argentata* nonrepetitive terminal regions were used as queries for the tBLASTx searches against nucleotide, transcriptome, and whole-genome shotgun databases at NCBI, and against a common house spider, *Parasteatoda tepidariorum*, database: Pt_spiderBase (http://www.e-celldev.jp/pt_spiderbase). Sequences with significant matches (*e*-value < 1e−05; Table S2) were visually inspected to confirm the presence of TuSp1 sequence motifs in adjoining repetitive regions and then trimmed to retain only terminal-encoding regions.

The N- and C-terminal-encoding regions were aligned separately because the majority of downloaded sequences were partial-length, containing only one terminal region (Table S2). For each region, amino acids from the translated sequences were aligned in Geneious v8.1.5 using the Clustal W algorithm and refined by eye (http://www.geneious.com; Thompson *et al.* 1994; Kearse *et al.* 2012). The amino acid alignments were then used as guides for aligning the nucleotides. The nucleotide alignments were analyzed using maximum likelihood gene tree construction with the GTRGAMMA model for nucleotide substitution and 10,000 bootstrap replicates as implemented in RAxML v8.2.8 (Stamatakis 2014). Resulting trees were visualized with FigTree v1.4.2 (http://tree.bio.ed.ac.uk/software/figtree/).

It should be noted that *A. bruennichi TuSp2* (synonym of *CySp2*; GenBank accession AB242145.1) was originally reported by Zhao *et al.* (2006) with an N-terminal region sequence that was divergent (69% pairwise nucleotide identity) from *A. bruennichi TuSp1* (synonym of *CySp1*; GenBank accession AB242144.1). Subsequently, Han and Nakagaki (2013) corrected the *A. bruennichi TuSp2* sequence and showed that the N-terminal-encoding regions of *A. bruennichi TuSp1* and *TuSp2* share 93% identity at the nucleotide level and 92% identity at the amino acid level. Our analyses were performed with the revised *A. bruennichi TuSp2* N-terminal-encoding region, which does not appear on GenBank. We took the revised sequence directly from the Han and Nakagaki (2013) publication.

### PCR survey of genomes

We verified the assemblies of the two shorter BAC clones and tested whether individual *Argiope* spider genomes have multiple *TuSp1* loci. To accomplish this, genomic DNA was extracted with the DNeasy Blood & Tissue Kit (QIAGEN) from single legs excised from each of two *A. argentata* individuals, one *A. aurantia* and one *A. trifasciata*.

The *A. aurantia* and the *A. trifasciata* genomic DNAs were the templates for PCR with degenerate primers designed to coamplify 5′ fragments of *TuSp1* and *TuSp1*ψ (Table S1). The 5′ fragment spanned part of the N-terminal-encoding region and the beginning of the repetitive region. Similarly, a second set of degenerate primers was designed to coamplify 3′ fragments of *TuSp1* and *TuSp1*ψ that encompassed the end of the repetitive region and most of the C-terminal-encoding region (Table S1).

PCR products of the expected size were purified (AccuPrep Gel Purification Kit, Bioneer) and Sanger sequenced in both directions on an Applied Biosystems 3730xl DNA Sequencer. Resulting chromatographs had overlapping peaks, indicative of heterogeneous amplification. Products were ligated into the pJET1.2 plasmid (Thermo Fisher Scientific) and transformed into TOP10 competent cells (Thermo Fisher Scientific). Individual colony inserts were PCR amplified using pJET1.2 forward and reverse sequencing primers (Table S1). Next, 186 PCR products with approximately equal sampling of 5′ and 3′ regions across species were purified (Table S3; AccuPrep PCR Purification Kit, Bioneer) and sequenced using nested pJET1.2 primers (Table S1).

### Diagnosis of PCR variants and phylogenetic analysis

Sequencher 3.1 (Gene Codes Corporation) was used to remove pJET2.1 vector from the raw sequencing reads and assemble contigs of the cloned and sequenced PCR products. Sequences for each region were aligned by species, and sequences with > 99% identities were considered to represent the same variant. Polymorphisms present in only one clone (unreplicated SNPs) were rare. These unreplicated SNPs were attributed to Taq polymerase error and ignored. If a single clone contained multiple unreplicated SNPs, it was discarded. Only six such clones (out of 186) were found. Therefore, each polymorphic site that we report is supported by at least two clones (Table S3). Variants were deposited on GenBank, accession numbers are in Table S3.

We aligned our *Argiope* PCR variants (Table S3) with corresponding *TuSp* regions from *Argiope* and other species within Araneidae that were available on GenBank on March 31, 2017 (Table S2). The 5′ and 3′ gene regions were analyzed separately. Tree construction and visualization were done as described above.

### Data availability

All sequence data are available on GenBank, and the accession numbers are listed in Table S2 and Table S3. Table S1 provides all primer sequences. Table S2 defines the sequences shown in Figure 3 and Figure 4 and provides accession numbers. Table S3 shows the number of clones supporting each PCR variant in each species and region, and provides accession numbers. File S1 contains the output from MultiPipMaker alignment of our BAC clone inserts, and alignments for the maximum likelihood analyses performed for Figure 3 and Figure 4.

## Results and Discussion

### A. argentata has both a tubuliform spidroin 1 gene and pseudogene

**Figure 1:**
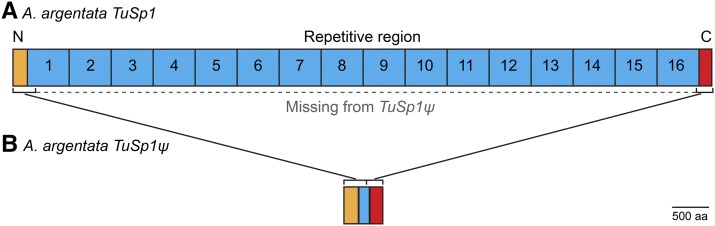
*A. argentata TuSp1* and pseudogene *TuSp1*ψ differ greatly in length, but have regions that can be locally aligned with high nucleotide identity (brackets; 5′ region has 92% pairwise nucleotide identity and 3′ region has 88% pairwise nucleotide identity). Gene regions shown to scale, scale bar lower right. (A) *TuSp1*. Repeats are numbered from 5′ to 3′. Most of the repetitive region is missing from *TuSp1*ψ (dotted line). (B) Pseudogene *TuSp1*ψ. Alignable regions are connected to corresponding region in *TuSp1*. aa, amino acids

**Figure 2:**
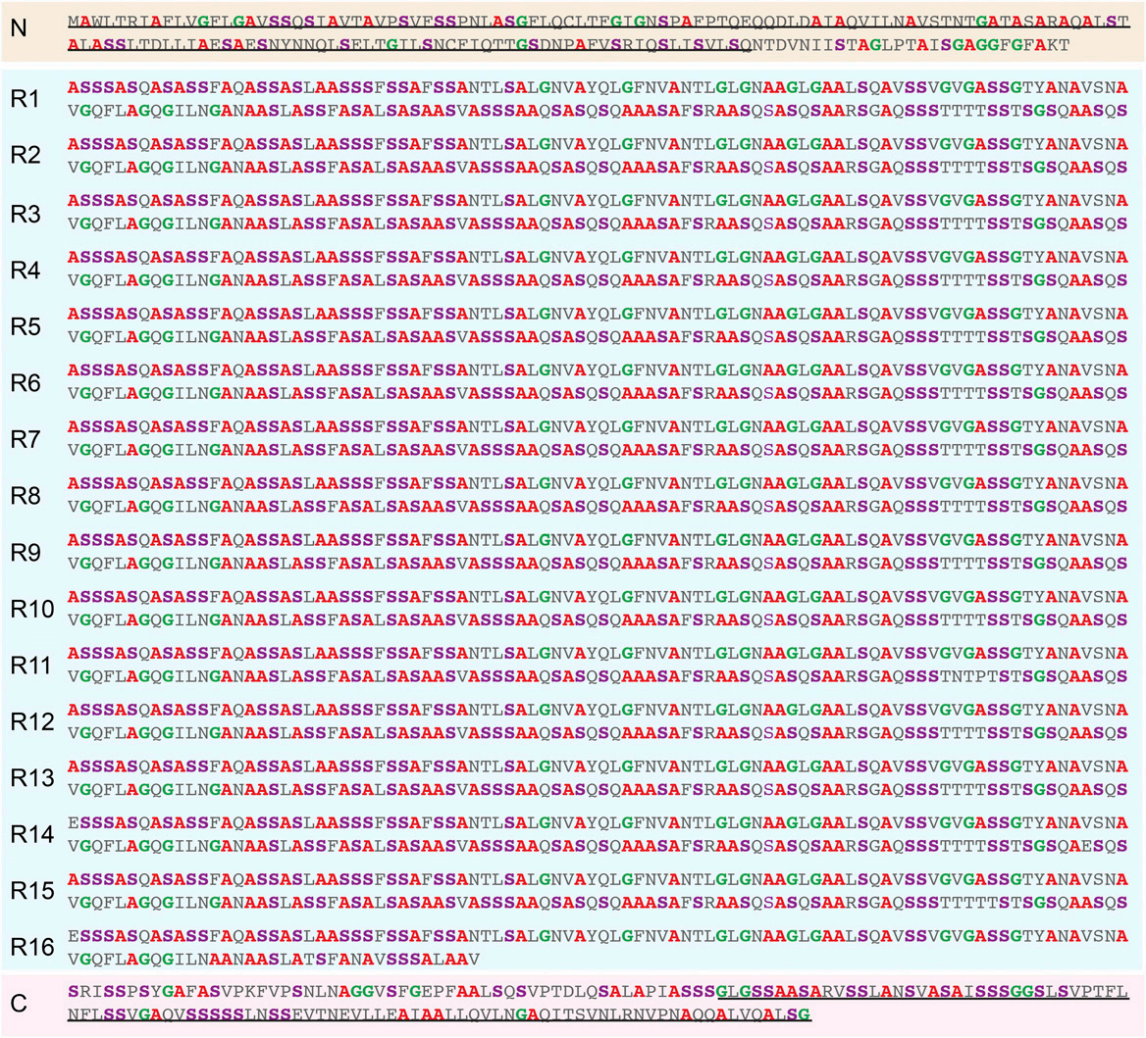
Translation of the complete *A. argentata TuSp1*, showing N-terminal, repetitive, and C-terminal regions. Conserved portions of the N- and C-terminal regions that are diagnostic for the spidroin family are underlined (Garb *et al.* 2010; Rising *et al.* 2006). Repeats are numbered as in Figure 1. Left: N = N-terminal region, R = repeat, and C = C-terminal region. Right: amino acids common to silk proteins (alanine, glycine, and serine) are highlighted for emphasis.

We sequenced BAC clones that contained genomic DNA inserts from the spider *A. argentata*. Assembly of the sequencing reads resulted in three contigs. The longest contig was ∼79 kb and contained a full-length gene, *Tubuliform Spidroin 1* (*TuSp1*; clone ID 31A22; accession # MF962652; Figure 1A, Figure 2, and Figure S1 in File S1). The two other contigs were ∼42 kb (clone ID 4G13) and ∼70 kb (clone ID 02O08) in length. The ∼42 kb contig was nested within the ∼70 kb contig; thus, the clones represented the same genomic region. Because the ∼42 kb contig shared 100% nucleotide identity with the ∼70 kb contig, all subsequent analyses were done with the longer contig. This genomic region was found to contain a pseudogene, *Tubuliform Spidroin 1*ψ (*TuSp1*ψ; accession # MF962653; Figure 1B).

To search for regions of similarity between the ∼79 and ∼70 kb assembled BAC clones, we used MultiPipMaker (Schwartz *et al.* 2000). As expected, the regions harboring *TuSp1* or *TuSp1*ψ were identified as similar. There were only six other areas with > 70% identity between the BAC clones. However, these areas were short, between ∼20 and 420 bp, and none had significant (*e*-value < 1e−05) BLAST matches to the NCBI nr database (Figure S1 in File S1). The dissimilarity of sequence outside of *TuSp1* and *TuSp1*ψ confirmed that these loci are in different genomic locations and are thus paralogs.

We searched for common eukaryotic promoter region sequences and found the TATAAA motif (TATA-box) ∼60 bp upstream of the predicted *TuSp1* start codon (Neural Network Promoter Prediction score 0.76; www.fruitfly.org/seq_tools/promoter.html; Reese 2001). We expected to find the TATA-box motif, as it has previously been found upstream of other spidroin genes in *A. argentata* and other spider species (Motriuk-Smith *et al.* 2005; Ayoub *et al.* 2007; Gaines and Marcotte 2008; Chaw *et al.* 2017; Vienneau-Hathaway *et al.* 2017). We also expected to find the CACG motif upstream of *A. argentata TuSp1*, as this motif was identified within ∼30 bp upstream of the TATA box and ∼80 bp upstream of the putative start codon in aciniform, flagelliform, major ampullate, minor ampullate, and pyriform spidroin genes (Motriuk-Smith *et al.* 2005; Ayoub *et al.* 2007; Chen *et al.* 2012; Chaw *et al.* 2017). However, we did not find the CACG motif, scanning as far as 1 kb upstream of the predicted start codon of *TuSp1*. The absence of CACG from *A. argentata TuSp1* suggests that *A. argentata TuSp1* may have a different regulatory mechanism compared to other spidroin genes.

We did not find any common promoter regions upstream of *TuSp1*ψ, which is consistent with it being a pseudogene. The absence of the TATA box motif is likely due to accumulated mutations after gene duplication. Alternatively, the lack of common promoter regions and the dissimilarity in the flanking regions of *TuSp1* and *TuSp1*ψ could indicate that *TuSp1*ψ arose through a recent retrotransposition event. However, we did not find other typical characteristics of pseudogenes resulting from retrotransposition, such as a 3′ poly-A tract or flanking repeats (Vanin 1985; Mighell *et al.* 2000).

### A. argentata TuSp1 and TuSp1ψ

*TuSp1* is a single-exon gene that is 9468 bp from start to stop codons (Figure 1A). This complete gene enhances knowledge of *A. argentata TuSp1*, which was previously known only from fragments (Garb and Hayashi 2005; Garb *et al.* 2010). *TuSp1* encodes short, nonrepetitive, N- and C-terminal regions that flank a large, central domain composed of 16 tandemly arrayed repeats. Fifteen of the repeats are exactly 540 bp in length, and the final repeat is 375 bp due to a truncation toward the 3′ end, as the gene transitions from the repetitive to the C-terminal-coding region (Figure 2).

The predicted *A. argentata* TuSp1 protein is 3156 amino acids. Typically, silk proteins are rich in alanine, glycine, and serine. *A. argentata* TuSp1 is abundant in alanine (26%) and serine (28%) but has much less glycine (8%; Figure 2). The 16 repeats have > 98% average pairwise identities at the nucleotide and amino acid levels. Dramatically, the first 10 repeats have 100% nucleotide identity. Thus, the arrayed *A. argentata TuSp1* repeat units are remarkably homogenized, depauperate in even silent nucleotide substitutions.

The pseudogene, *TuSp1*ψ, is 1357 bp (Figure 1). *TuSp1*ψ has a premature stop codon at 421–423 due to a frame shift, with recognizable spidroin sequence downstream of this frame shift. A coding region of 423 bp, or even 1357 bp, would be shorter than any currently annotated complete spidroin gene and shorter than many partial length spidroin cDNAs. To investigate the possibility of an assembly error resulting in a chimeric sequence with false premature stops or a cloning error during library construction, we amplified the entire locus from two *A. argentata* individuals (independent amplification from each individual; these were different individuals than those represented in the BAC library), followed by direct sequencing of the ∼1.4 kb PCR products. The PCR-amplified *TuSp1*ψ were identical in nucleotide sequence between individuals and also with BAC clone *TuSp1*ψ. Hence, we verified that *TuSp1*ψ is a pseudogene and not an artifact of cloning or assembly. This is the first reported *TuSp1* pseudogene; however, pseudogenes for two other spidroin types have previously been described (Ayoub and Hayashi 2008; Vienneau-Hathaway *et al.* 2017).

To compare *TuSp1* with *TuSp1*ψ, we aligned the sequences, including the insertion of gaps to account for two frame shifts in *TuSp1*ψ, thereby eliminating all premature stop codons (Figure 1). We found that *TuSp1* and *TuSp1*ψ have an alignable 5′ segment that codes for the conserved spidroin N-terminal region and 286 bp of the first *TuSp1* repeat (R1), and an alignable 3′ segment that codes for 84 bp of the last *TuSp1* repeat (R16) and the conserved spidroin C-terminal region. The 5′ and 3′ segments of *TuSp1* align to the corresponding regions in *TuSp1*ψ with 92 and 88% pairwise nucleotide identity, respectively (Figure 1, brackets).

There are several possible explanations for the high nucleotide similarities between the alignable segments of *TuSp1* and *TuSp1*ψ (Figure 1). One explanation is that *TuSp1* duplicated recently and that only a modest amount of divergence has occurred between the copies. Another possibility is that *TuSp1* duplicated in the distant past and that the two loci have remained homogenized through selection and/or gene conversion. Selection alone seems unlikely given the general lack of variation even in third-codon positions.

Whether the high sequence similarity between the shared sequence of *TuSp1* and *TuSp1*ψ is due to a recent duplication event (*i.e.*, little time for sequences to diverge) or homogenization of ancient gene copies, a different mechanism is needed to explain the loss of nearly all of the *TuSp1* repetitive region from *TuSp1*ψ. We suggest that the deletion of repeats occurred via unequal recombination mediated by the high sequence identity among tandem repeats (R1–R16, Figure 2). Given the internally repetitive sequence of spidroins, unequal crossing over events during recombination are likely (Beckwitt *et al.* 1998; Hayashi and Lewis 2001; Ayoub *et al.* 2007; Zhao *et al.* 2010). We expect that dramatic losses of repeats within repetitive regions would be strongly selected against in functional spidroin genes. However, a pseudogenized spidroin gene is released from selection, which explains the frame shifts and premature stop codons in *TuSp1*ψ, and the erosion of repeats from *TuSp1*ψ.

### A. argentata TuSp1 and TuSp1ψ group with other TuSp1 sequences from Araneidae

**Figure 3:**
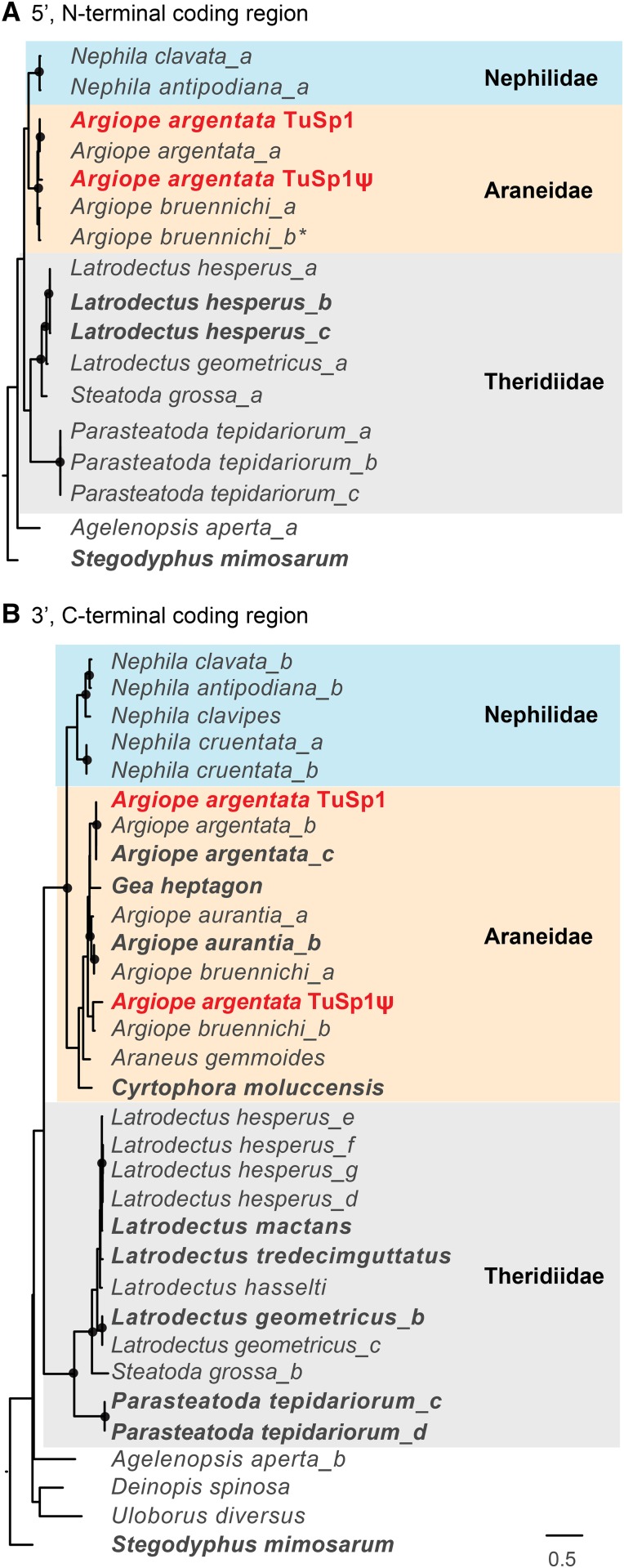
Maximum likelihood analyses of terminal regions of *A. argentata TuSp1* and *TuSp1*ψ with annotated *TuSp1* sequences. (A) N-terminal-encoding region. (B) C-terminal-encoding region. Sequence names, descriptions, and accession numbers are in Table S2. Trees are rooted with the velvet social spider, *S. mimosarum*. Bold font indicates sequences from genomic DNA, plain from cDNA. Asterisk indicates the corrected sequence of the N-terminal-encoding region of *A. bruennichi TuSp2* (Han and Nakagaki 2013). Black circles indicate nodes with > 80% bootstrap support over 10,000 replicates. Scale bar is substitutions per site.

We reconstructed the phylogenetic relationships of our *A. argentata TuSp1* and *TuSp1*ψ with other tubuliform spidroin genes. Our maximum likelihood analyses included all published *TuSp1* sequences (as of March 31, 2017) and were rooted with *TuSp1* from the social velvet spider *Stegodyphus mimosarum*, a species outside of the Araneoidea and Deinopoidea (accession numbers in Table S2; alignments in Figure S2 and Figure S3 in File S1). We recovered monophyletic groupings of araneoid (which includes Araneidae, Nephilidae, and Theridiidae) tubuliform spidroin N-terminal- and C-terminal-coding regions (Figure 3). Looking more closely at the resolution of sequences within the araneoid clade of sequences, *A. argentata* is a member of the Araneidae, and in both the N- and C-terminal-coding region trees, the sequences from the Araneidae form a clade (*Araneus gemmoides*, *Argiope* spp., *Cyrtophora moluccensis*, and *Gea heptagon*). Furthermore, the sister group relationship of Araneidae and Nephilidae agrees with recent phylogenomic studies of spiders (Dimitrov *et al.* 2012; Bond *et al.* 2014; Garrison *et al.* 2016; Wheeler *et al.* 2016).

Our complete *A. argentata TuSp1* groups with partial-length *A. argentata* cDNA sequences (cDNA sequences in plain font, genomic DNA sequences in bold; Figure 3). This grouping supports the notion that our complete *TuSp1* is functional. The relationship of *A. argentata TuSp1* with *A. argentata TuSp1*ψ is unclear. The analysis of N-terminal-coding regions allies the two sequences into a clade (Figure 3A). This relationship suggests that gene duplication may have occurred independently within the *A. argentata* and *A. bruennichi* lineages. Alternatively, this relationship could indicate that the gene duplication occurred in a common ancestor of the two species with the gene copies homogenizing within each species. In contrast, the C-terminal coding regions of *A. argentata TuSp1* groups with other *Argiope* and *Gea TuSp1*, whereas the *A. argentata TuSp1*ψ allies with *A. bruennichi TuSp2* (*A.bru_b*; Figure 3B). This grouping of *TuSp1*ψ with *A. bruennichi TuSp2* rather than with the *A. argentata TuSp1* sequences implies that the *TuSp* duplication event occurred in a common ancestor of the *Argiope* and *Gea* lineage.

### Evidence for two TuSp1 loci in focal Argiope species

**Figure 4:**
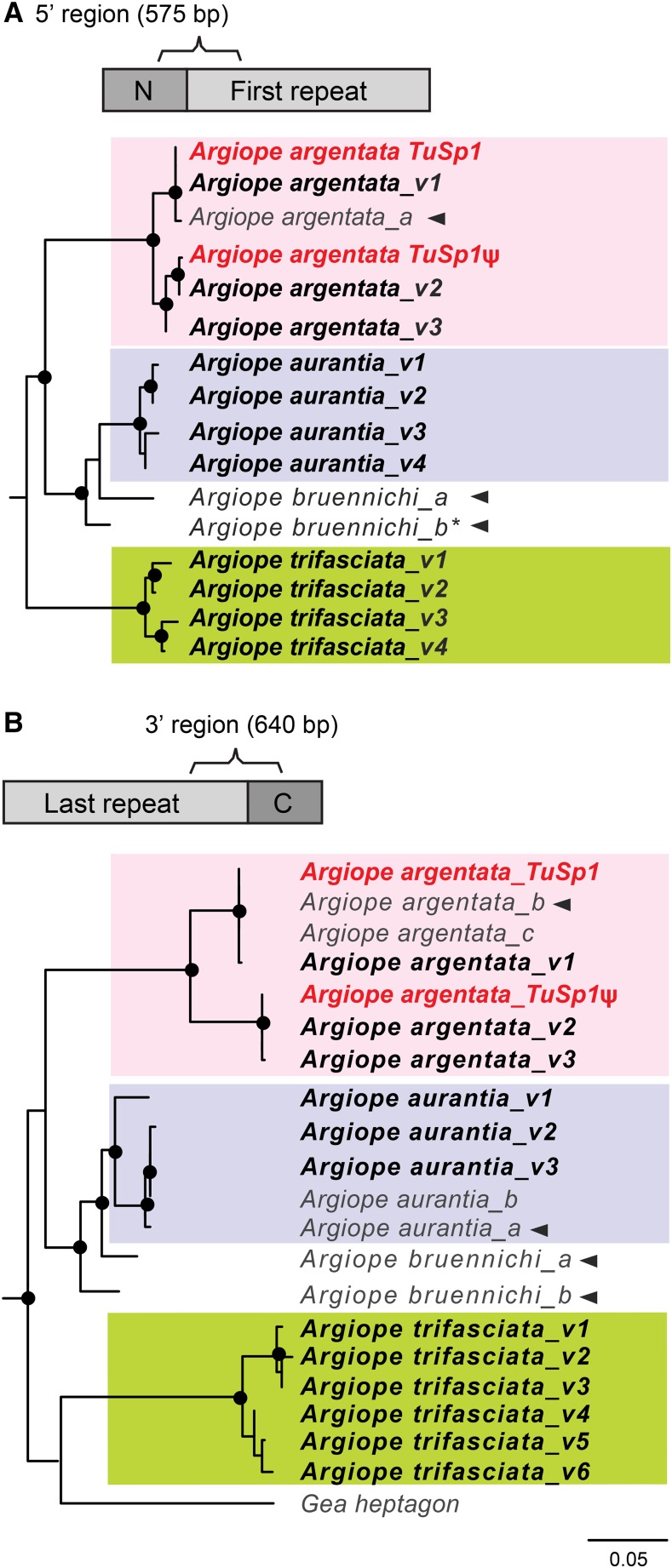
Clustering of *TuSp1* PCR variants sequenced from *Argiope* spp. with available *Argiope TuSp1* sequences. Total number of aligned nucleotides shown in parenthesis, bracket indicates the portion of the N-terminal, C-terminal, and repetitive region encoded by the nucleotides in each analysis. (A) 5′ region. (B) 3′ region. *TuSp1* 5′ and 3′ region variants (v; numbered, bold) group into two distinct clades within each species (shaded boxes). The 5′ region tree is midpoint rooted whereas the 3′ region tree is rooted with *C. moluccensis* (data not shown). Sequence names, descriptions, and GenBank accession numbers are in Table S2 and Table S3. Asterisk indicates that analyses were done with the corrected *A. bruennichi TuSp2* (Han and Nakagaki 2013). Black circles indicate nodes with > 80% bootstrap support over 10,000 replicates. Arrowheads indicate cDNA sequences. Scale bar indicates substitutions per site.

We PCR screened the genomic DNA of *A. argentata*, *A. aurantia*, and *A. trifasciata* individuals for the presence of *TuSp1* gene duplicates. Degenerate primers were used to amplify 5′ fragments of *TuSp1* loci and a second set of degenerate PCR primers were used to amplify 3′ fragments. The 5′ fragments included sequences that encoded portions of the N-terminal regions and the immediately adjacent repetitive regions (Figure 4A). Similarly, the 3′ fragments encompassed part of the last repetitive and C-terminal-encoding regions (Figure 4B). Each PCR reaction amplified a mixture of amplicons, and thus, the PCR products were cloned to separate individual amplicons, which were then sequenced. From these sequences, variants were identified (Table S3). For simplicity, variants for each region within a species were numbered sequentially (v1, v2, and v3, etc.).

Because we surveyed genomic DNA from individual spiders, we were able to infer the minimum number of *TuSp* loci in each species. There are at least two loci in *A. argentata* (three variants each for 5′ and 3′ regions; spiders are diploid) and at least two loci also in *A. aurantia* (four variants for the 5′ region and three variants for the 3′ region), *A. trifasciata* is slightly richer with at least three loci (four variants for the 5′ region but six for the 3′ region; Figure 4 and Table S3).

Maximum likelihood analyses placed the *TuSp* 5′ region variants, and separately the 3′ region variants, from each of our focal species into well-supported, species-specific groups (Figure 4, orange, blue, and green clades). The monophyletic grouping of the variants by species is consistent with ongoing homogenization of *TuSp* loci within species. This is a more parsimonious explanation than the scenario of each species having independently duplicated *TuSp1* gene copies. However, our ability to detect multiple variants within a species suggests that the homogenization is imperfect. The observed variation could be due to a time lag in homogenization or diversifying selection between gene copies, perhaps due to differential expression patterns.

Each species-specific grouping of PCR variants can be subdivided into two or more subclades (Figure 4 and alignments in Figure S4 and Figure S5 in File S1). For *A. argentata*, the 5′ and the 3′ region PCR variants can each be assigned to different subclades. One subclade includes the BAC clone-derived *A. argentata TuSp1* and the other includes the *A. argentata TuSp1*ψ (red font, Figure 4). The 5′ region PCR variants from *A. aurantia* also are divided into two subclades. The *A. aurantia* 3′ region PCR variants have one divergent variant outside of a subclade containing highly similar variants plus conspecific sequences from GenBank. The 5′ and 3′ region PCR variants from *A. trifasciata* also group into one of two distinct subclades that are sister to each other. We posit that the species-specific *TuSp1* subclades indicate distinct loci. Additional genomic sequencing of *Argiope* species is needed to confirm that each *TuSp1* locus is in a unique genomic location.

A few of the *TuSp* sequences from GenBank that we incorporated into our analyses are cDNAs (Figure 4, arrowheads), providing evidence for which of our variants correspond to expressed genes. BAC clone *A. argentata TuSp1*, and PCR variants *A. argentata* 5′ *v1* and *A. argentata* 3′ *v1*, appear to be functional because they group with *A. argentata* cDNAs (Figure 4). Similarly, *A. aurantia* 3′ *v2* and *v3* group with an *A. aurantia* cDNA and thus are likely to be expressed (Figure 4B). On the contrary, we found no evidence for expression of *A. aurantia* 3′ *v1* because it does not group with the *A. aurantia* cDNA (Figure 4B). We also did not find evidence for expression of any of the *A. aurantia* 5′ region variants or any of the *A. trifasciata* variants. Whether any of these genes are pseudogenes requires further investigation.

### Conclusions

We characterized two large genomic regions containing *TuSp1* loci in *A. argentata* and discovered multiple *TuSp1* loci in other *Argiope* spiders. One of the *A. argentata* genomic regions encompassed a full-length *TuSp1* gene and the other a *TuSp1*ψ pseudogene (Figure 1). The *A. argentata TuSp1* gene encodes a predicted protein of 293 kDa. This protein is dominated by 16 repeats that are both long (∼180 amino acids) and exceptionally homogenized (98% average pairwise amino acid and nucleotide identity; Figure 2). This astonishing identity among the full complement of *A. argentata* TuSp1 repeats is consistent with hypotheses of intragenic concerted evolution among spidroin repetitive regions (*e.g.*, Hayashi and Lewis 2000, Chaw *et al.* 2017).

In contrast to the length of *A. argentata TuSp1*, *A. argentata TuSp1*ψ is extremely truncated with only 1357 bp (*A. argentata TuSp1* is 9468 bp). The difference in length between *A. argentata TuSp1* and *TuSp1*ψ can be entirely accounted for by a loss of all but a fraction of the repetitive region (Figure 1). We hypothesize that *TuSp1*ψ recently underwent pseudogenization based on its high pairwise nucleotide identity to the corresponding 5′ (92%) and 3′ (88%) regions of *TuSp1* (Figure 1). Furthermore, nonreciprocal recombination facilitated by the tandemly arrayed repeats is a likely mechanism for the missing repetitive sequence in *TuSp1*ψ (*e.g.*, Beckwitt *et al.* 1998, Hayashi and Lewis 2001, Ayoub *et al.* 2007, Zhao *et al.* 2010), with the loss of repeats having little or no fitness consequence. In a functional spidroin gene, repetitive regions directly contribute to silk mechanical properties (*e.g.*, Gatesy *et al.* 2001, Li *et al.* 2017), thus massive loss of repeats is expected to be strongly selected against. *A. argentata TuSp1*ψ provides insights into how spidroin loci evolve by showing the fate of a locus that is no longer under selection.

Maximum likelihood analyses of the N-terminal-coding region of *TuSp* from multiple species resulted in species-specific clades. This pattern is consistent with intergenic homogenization occurring within species. However, our ability to detect multiple copies of *TuSp1* in each species suggests that intergenic homogenization of the terminal regions is not as complete as intragenic homogenization of *TuSp1* repeats within a species (Figure 2 and Figure 3; Garb and Hayashi 2005). In fact, phylogenetic analysis of the C-terminal-coding regions of *TuSp* from multiple species grouped *A. argentata TuSp1*ψ and *A. bruennichi TuSp2* with each other instead of their corresponding, conspecific sequences (Figure 3B). Less similarity between the terminal domains of the functional and pseudogenized *A. argentata* sequences is expected, because the pseudogene is released from selection and therefore can accumulate mutations.

Adding repetitive region sequence and more terminal regions to the phylogenetic analyses resulted in species-specific groups. By PCR screening of individual genomic DNAs, we showed that, in addition to *A. argentata* and *A. bruennichi*, *A. aurantia* and *A. trifasciata* also have multiple *TuSp1* loci (Figure 4 and Table S2). Phylogenetic analyses of the variant fragments, which were 5′ or 3′ regions that included partial N- or C-terminal-coding sequence plus partial repetitive regions, clustered the variants by species (Figure 4). Indeed, inclusion of repetitive region sequence grouped *A. argentata TuSp1*ψ with other *A. argentata TuSp1* variants (Figure 4B) instead of with *A. bruennichi TuSp2* (Figure 3B). Likewise, *A. bruennichi TuSp1* and *TuSp2* grouped with each other and with *A. aurantia TuSp1* variants (Figure 4), which is consistent with the sister group relationship of *A. aurantia* and *A. bruennichi* recovered in a molecular systematic study of *Argiope* (Agnarsson *et al.* 2016). Clustering of variants by species could be explained by gene duplications having taken place independently within each species. This scenario implies that gene turnover is occurring frequently and convergently across species. A simpler evolutionary explanation is that multiple *TuSp1* gene copies were present in a distant *Argiope* ancestor, with subsequent divergence between species and homogenization of gene copies within species, most dramatically in the repetitive regions.

Less homogenization among terminal domains may reflect differences in functional constraints on the terminal and repetitive regions. In contrast to the importance of the repetitive region for silk mechanical properties, spidroin terminal domains are involved in the assembly of multiple spidroin proteins into a fiber and in the transition of the proteins from suspension in a liquid to a solid dry fiber (*e.g.*, Ittah *et al.* 2007, Hedhammar *et al.* 2008, Kronqvist *et al.* 2014). Greater variation among *TuSp1* terminal regions may still result in successful fiber assembly, whereas the repeats may need to be nearly identical for fibers to function as effective egg case wrapping.

## Supplementary Material

Supplemental material is available online at www.g3journal.org/lookup/suppl/doi:10.1534/g3.117.300283/-/DC1.
