# Supplementary material for: Egg Case Silk Gene Sequences from Argiope Spiders: Evidence for Multiple Loci and a Loss of Function Between Paralogs
Source: G3 (Bethesda). 2017 Nov 10;8(1):231–8. doi: 10.1534/g3.117.300283 (PMC5765351; doi:10.1534/g3.117.300283)
Supplement: Supplementary file 1 [file 231TableS1.docx]

**Table S1**. Primer sets for BAC library screening, pseudogene verification, amplification of multiple *TuSp* loci, and pJET plasmid sequencing. Primers shown in 5’ to 3’ orientation.

| **Primer** | **Forward** | **Reverse** |
| --- | --- | --- |
| BAC library screening | GCTTTCTCCAGTGYCTTCTC | GCTTGTGCGAAGGAAGAGGCACT |
| Pseudogene sequencing | AGAACAGGTGGCTTATCAGTCAAT | GGCAATTATTCCCACAAAAACTGT |
| TuSp N-terminal, degenerate | CAACARGRYTTGGATGCCATTGC | CTGCATTGGARASGRCATTAGCG |
| TuSp C-terminal, degenerate | TTTCTCMAGTGCCTTCTCYTCGGC | ASCYCCRTTRAKRACTTGCAAGAG |
| pJET 1.2 sequencing | CGACTCACTATAGGGAGAGCGGC | AAGAACATCGATTTTCCATGGCAG |
| pJET 1.2 nested sequencing | CCAGATCTTCCGGATGGCTC | GATTTTCCATGGCAGCTGGAG |
