# Supplementary material for: Egg Case Silk Gene Sequences from Argiope Spiders: Evidence for Multiple Loci and a Loss of Function Between Paralogs
Source: G3 (Bethesda). 2017 Nov 10;8(1):231–8. doi: 10.1534/g3.117.300283 (PMC5765351; doi:10.1534/g3.117.300283)
Supplement: Supplementary file 4 [file 231FileS1.docx]

Figure S1. Output from MultiPipMaker. Top: Schematic of BAC clone 31A22, which contains the complete coding region of *Argiope argentata TuSp1,* showing regions with >70% nucleotide identity (pink and grey bars) to BAC clone 020O8, the clone that contains *Argiope argentata TuSp1*ψ. As expected, the region with the *TuSp1* full-length gene (*TuSp1*) has regions with high nucleotide identity (grey bars). MultiPipMaker can align a given region more than once, and most of the regions aligned in the *TuSp1* region are a truncated repeat from *TuSp1*ψ mapping iteratively to the repeats in *TuSp1*. For an accurate alignment of *TuSp1* to *TuSp1*ψ, please see Figure 2 and Figs. S2, S3. The lengths (in base pairs) of the six regions with high nucleotide identity outside of *TuSp1* (pink bars) are shown in the table. Bottom: Nucleotide alignments of the six regions with >70% nucleotide identity outside of *TuSp1.* Sequence from clone 31A22 is above sequence from clone 020O8.


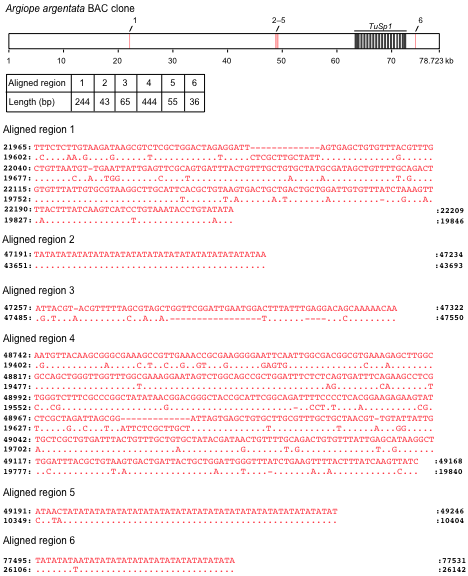
Figure S2. Nucleotide alignment of N-terminal encoding regions from *Argiope argentata TuSp1* and *TuSp1*ψ characterized from BAC clones (bold) and previously characterized tubuliform spidroins. A.bru_b* sequence includes corrections reported by Han and Nakagaki (2013). Complete sequence names and GenBank accession numbers are in Table S2. Missing (unknown) sequence indicated by “?”, and alignment gaps by dashes.

**A.arg_TuSp1** ATGGCTTGGC TCACTAGAAT TGCGTTCCTT GTAGGCTTTC TGGGAGCAGT 50

**A.arg_TuSp1ψ** ATGATCTCGT TCACAACAGT AGCTCTCCTA ATTGGGCTTT TAGGACCAGT

A.arg_a ?????????? ?????????? ?????????? ?????????? ??????????

A.bru_a ATGGTTTGGC TCACTAGCAT AGCGTTCCTC GTAGGCTTTC TGGGAGCAGT

A.bru_b* ATG---TGGT TCACTACAGT AGCGTTCCTC TTATGCCTTT TAGGAGCAGT

N.ant_a ATGGTTTGGC TGACGAGTAT CGCGTTTATT GTGACTCTTT TAGGAGCACA

N.clava_a ATGGTTTGGC TGACAAGCAT AGCGTTTGTT GTGGCTCTTT TAGGAGCACA

P.tep_a ATGGTGTGGT TTCCTTCCAT TGCCTTCTCA ATAACCCTTT TGGGAGGGCT

P.tep_b ATGGTGTGGT TTCCTTCCAT TGCCTTCTCA ATAACCCTTT TGGGAGGGCT

P.tep_c ATGGTGTGGT TTCCTTCCAT TGCCTTCTCA ATAACCCTTT TGGGAGGGCT

L.hes_a ATGGTTTGGT TGACAAGTAC TGTGCTCCTA GCATCCCTTC TGGGAACACT

L.hes_b ATGGTTTGGT TGACAAGTAC TGTGCTCCTA GCATCCCTTC TGGGAACACT

L.hes_c ATGGTTTGGT TGACAAGTAC TGTGCTCCTA GCATCCCTTC TGGGAACACT

L.geo_a ATGGTTTGGT TGACAAGTAC TGTGCTCCTA GCATCCCTTC TGGGAACACT

S.gro_a ATGATTTGGT TGACAAGTAC AGTACTCCTA GTATCCCTGT TGGGAACACT

A.ape_a ATGGTTTGGA TAACTAGCAT AGCATGTCTG TTTACCTTTA TAGCCACATT

S.mim ATGGTTTGGT TACCTACCCT ACCGTATATA TTTATCCTTC TGGGAGCATT

**A.arg_TuSp1** GTCTTCTCAG ---------- --------TC AATTGCAGTT ---------- 100

**A.arg_TuSp1ψ** AACGTCCCAG ---------- --------TC AGATGGAGTT ----------

A.arg_a ?????????? ?????????? ?????????? ????GCAGTT ----------

A.bru_a GTCGTCTCAG ---------- --------TC AGTTGCAGTT ----------

A.bru_b* GTCGTCACAA ---------- --------TC AGTTGCAGTT ----------

N.ant_a ATACGACATA GTGACTGCGC AGGCAATTTC AGTTGCA--- ----------

N.clava_a ATACGACATC GTGACTGCTC AGGCAATTTC AGTTGCA--- ----------

P.tep_a GGCTTCCCTT ATCACAGGGG CTCCTTTGAC ATCTGCAAAT ----------

P.tep_b GGCTTCCCTT ATCACAGGGG CTCCTTTGAC ATCTGCAAAT ----------

P.tep_c GGCTTCCCTT ATCACAGGGG CTCCTTTGAC ATCTGCAAAT ----------

L.hes_a T--------- ------GGAC TTCCAGCCAA TAGTTTA--- ---TCAGGAG

L.hes_b T--------- ------GGAC TTCCAGCCAA TAGTTTA--- ---TCAGGAG

L.hes_c T--------- ------GGAC TTCCAGCCAA TAGTTTA--- ---TCAGGAG

L.geo_a T--------- ------GGAC TTCCAGCCAA CAGTTTCTCA TCATCAGGAG

S.gro_a T--------- ------GCAA ATCCAGCCAT CTCTTACTCT TCATCGCAAG

A.ape_a TGGCAGTCAA GTTGGAGCAC AAGGAACT-- -------TCT TTTGCATCAT

S.mim ATATGACTCG ACGTACGCAC AGACAAC--- ---------- ----------

**A.arg_TuSp1** --------AC CGCCGTTCCA TCAGTCTTCA GTAGTCCGAA TTTAGCAAGC 150

**A.arg_TuSp1ψ** --------AT CGCATTTCCC TCAGTTTTCA GCAATCCGAA ATTAGCCTAC

A.arg_a --------AC CGCCGTTCCA TCAGTCTTCA GTAGTCCGAA TTTAGCAAGC

A.bru_a --------AC CGCCGTTCCT TCAGTCTTCA GCAGTCCAAA TTTGGCCAGC

A.bru_b* --------AC CGCCGTTCCC TCAGTCTTCA GCAGTCCAAA TTTGGCCAGC

N.ant_a --------AC TTCTGTCCCA TCAGTGTTCA GTAGCCCTAG CCTTGCAAGT

N.clava_a --------AC TCCTGTCCCA TCAGTGTTCA GTAGCCCTAG CCTTGCGAGT

P.tep_a --------GA CGCTTCGCAG ATAGTATTTA GCAATCCGCA AATAGCAAGG

P.tep_b --------GA CGCTTCGCAG ATAGTATTTA GCAATCCGCA AATAGCAAGG

P.tep_c --------GA CGCTTCGCAG ATAGTATTTA GCAATCCGCA AATAGCAAGG

L.hes_a TT------TC AGCCTCGGTC AATATTTTCA ATAGCCCCAA TGCAGCAACT

L.hes_b TT------TC AGCCTCGGTC AATATTTTCA ATAGCCCCAA TGCAGCAACT

L.hes_c TT------TC TGCCTCGGTC AATATTTTCA ATAGCCCCAA TGCAGCAACT

L.geo_a TTTCTGTCTC AAACGCGGTC AATGTTTTCA ATAGTCCCAG TGCAGCAACT

S.gro_a CTTCAGCCTC AAGCGCAGTC AATGTATTCA CTAGTCCTAG TGCAGCAAGT

A.ape_a CAGCAACAGC AGGAATACGA AATATATTTG GAAATCCAAA CACTGCAAAT

S.mim -AGCAGGTGG AGGGATAAAT AGAGTATTCG GAAATCCTTT TATCGCTAAA

**A.arg_TuSp1** GGCTTCCTTC AGTGCCTCAC ATTTGGAATC GGAAATTCTC CCGCATTTCC 200

**A.arg_TuSp1ψ** GGGTTCCTCC AATGTCTGAC ATTTGGAATC GGAAATTCTC CCGCATTTCC

A.arg_a GGCTTCCTTC AGTGCCTCAC ATTTGGAATC GGAAATTCTC CCGCATTTCC

A.bru_a GGCTTCCTTC AATGTCTCAC ATTTGGAATC GGAAATTCCC CCGCATTTCC

A.bru_b* GGTTTCCTTC AGTGTCTCAC ATTTGGAATC GGAAATTCTC CCGCATTTCC

N.ant_a GGTTTCCTTG GATGTCTCAC AACTGGTATT GGACAATCTC CAGATTTCCC

N.clava_a GGTTTCCTTG GATGCCTCAC AACTGGTATT GGACTATCTC CAGCTTTCCC

P.tep_a GAGTTTCTTC AGTGCCTTAC GAGCAGCATC GGAGCTTCTC CAGACTTTCC

P.tep_b GAGTTTCTTC AGTGCCTTAC GAGCAGCATC GGAGCTTCTC CAGACTTTCC

P.tep_c GAGTTTCTTC AGTGCCTTAC GAGCAGCATC GGAGCTTCTC CAGACTTTCC

L.hes_a AGTTTCCTAA ACTGTCTTAG ATCTAATATA GAATCTTCTC CAGCATTCCC

L.hes_b AGTTTCCTAA ACTGTCTTAG ATCTAATATA GAATCTTCTC CAGCATTCCC

L.hes_c AGTTTCCTAA ACTGTCTTAG ATCTAATATA GAATCTTCTC CAGCATTCCC

L.geo_a AGTTTCCTAG AATGTCTTAC AACCAGTATA GAATCTTCTC CAATATTCCC

S.gro_a AGTTTTCTTC AATGTCTTAC ATCGAGTATA GGATCTTCTC CAGCATTTCC

A.ape_a AACTTCGTTG ACTGTCTTAA AGGAGGAATT CAGGCATCCC CAGCTTTCCC

S.mim ACATTTCTTC AGTGCCTGAC TTCAAGTATA GGATCATCAA AAGCCTTTCC

**A.arg_TuSp1** CACTCAAGAA CAACAAGATT TGGATGCCAT TGCTCAAGTG ATACTCAATG 250

**A.arg_TuSp1ψ** CACTCAAGAA CAACAGGGCT TGGATGCCAT TGCCCAAGTG ATACTCAATG

A.arg_a CACTCAAGAA CAACAAGATT TGGATGCCAT TGCTCAAGTG ATACTCAATG

A.bru_a TACTCAAGAA CAACAAGACT TGGATGCCAT TGCTCAGGTG ATACTCAATG

A.bru_b* TACTCAAGAA CAACAAGACT TGGATGCCAT TGCCCAGGTG ATACTCAATG

N.ant_a GTTTCAAGAA CAACAAGATT TAGATGACTT AGCACAAGTA ATTCTTTCGG

N.clava_a GTTTCAAGAA CAACAAGATT TAGATGACTT AGCCAAGGTA ATTCTCTCCG

P.tep_a CGCACAAGAG CAGTCAGAAA TAGAAACAAT TACGGAAGCC ATACTTTCCA

P.tep_b CGCACAAGAG CAGTCAGAAA TAGAAACAAT TACGGAAGCC ATACTTTCCA

P.tep_c CGCACAAGAG CAGTCAGAAA TAGAAACAAT TACGGAAGCC ATACTTTCCA

L.hes_a AATCCAAGAA CAGGCTGACT TAGATTCTAT CGCGGAAGTA ATTCTTTCAG

L.hes_b AATCCAAGAA CAGGCTGACT TAGATTCTAT CGCGGAAGTA ATTCTTTCAG

L.hes_c ATTCCAAGAA CAGGCTGACT TAGATTCTAT CGCGGAAGTA ATTCTTTCAG

L.geo_a GATTCAAGAA CGGGCTGATT TGGATTCTAT TGCAGAAGTC ATTCTTTCTG

S.gro_a TATCCAACAA CAGGATGATT TAGATTCTAT TGCAAAAGCC ATTCTTTCTG

A.ape_a ACGACAAGAA CAAGCTGATA TCCAGTCAAT TGCAAGTTCC ATACTTTCAG

S.mim TTCCCAAGAA CAAGATGACA TTGATGCTAT TGCAGGTGTA ATTCTCTCGG

**A.arg_TuSp1** CCGTTTCAAC AAATACTGGC GCCACAGCAT CAGCCAGAGC TCAAGCTTTA 300

**A.arg_TuSp1ψ** CCGTTTCAAG CAATACTGGC CCCACAGCAT CAGCCAGAGC TCAAGCTTTA

A.arg_a CCGTTTCAAC AAATACTGGC GCCACAGCAT CAGCCAGAGC TCAAGCTTTA

A.bru_a CTGTTTCAAG CAACACTGGC GCTACAGCAT CGGCCAGAGC TCAAGCTTTA

A.bru_b* CCGTTTCAAG CAACACTGGC GCCACAGCAT CGGCCAGAGC TCAAGCTTTA

N.ant_a CAGTAACCAG TAATACTGAC ACCTCAAAGT CAGCGAGAGC ACAAGCCTTG

N.clava_a CAGTAACCAG TAATACTGAC ACCTCAAAGT CAGCGAGAGC ACAAGCCTTG

P.tep_a CTGTATCAAA TTCGAATGAA CAATCAATTG AAACTAGGTC ACGAGCTGCA

P.tep_b CTGTATCAAA TTCGAATGAA CAATCAATTG AAACTAGGTC ACGAGCTGCA

P.tep_c CTGTATCAAA TTCGAATGAA CAATCAATTG AAACTAGGTC ACGAGCTGCA

L.hes_a ATGTATCCAG TGTGAATACC GCAAGCTCAG CAACA---TC TTTAGCGCTA

L.hes_b ATGTATCCAG TGTGAATACC GCAAGCTCAG CAACA---TC TTTAGCGCTA

L.hes_c ATGTATCCAG TGTGAATACC GCAAGCTCAG CAACA---TC TTTAGCGCTA

L.geo_a ATGTATCCAG TGTGAACAAT GCACGCGCAG CGACA---TC TTTAGCACTA

S.gro_a ATGTATCCAG CGTAAGCAGT GCACGCACAA CTGCAACGGC TCAAGCACTG

A.ape_a CTGGT----- -AATACTGCA ACAAAATCGA AAGCAATAGA ACAAGCTTTA

S.mim CTGTC---TC AAATTCAAAC TCCGTCTCCT CAGCTAGAGC AGAAGCTTTA

**A.arg_TuSp1** AGTACAGCGC TCGCATCCTC TCTCACAGAT CTCCTCATTG CAGAGTCGGC 350

**A.arg_TuSp1ψ** AGTACAGCGC TCGCATCTTC TCTCACAGAT CTGCTCATTG CAGAGTCGGC

A.arg_a AGTACAGCGC TCGCATCCTC TCTCACAGAT CTCCTCATTG CAGAGTCGGC

A.bru_a AGTACAGCGC TTGCATCTTC TCTGACAGAT CTGCTCATTG CAGAGTCGGC

A.bru_b* AGTACAGCGC TTGCATCTTC TCTGACAGAT CTGCTCATTG CAGAGTCGGC

N.ant_a AGCACTGCAT TAGCATCTTC CTTAGCCGAC CTACTGATTT CCGAATCAAG

N.clava_a AGCACTGCAT TAGCATCTTC CTTAGCCGAC CTACTGATAT CCGAATCAAG

P.tep_a AATATTGCGT TAGCTTCATC ATTAGCTGGC TTGTTAGCGA GTGATTCAAA

P.tep_b AATATTGCGT TAGCTTCATC ATTAGCTGGC TTGTTAGCGA GTGATTCAAA

P.tep_c AATATTGCGT TAGCTTCATC ATTAGCTGGC TTGTTAGCGA GTGATTCAAA

L.hes_a AGTACTGCTT TAGCATCGTC GTTGGCTGAA CTACTTGTCA CTGAATCAGC

L.hes_b AGTACTGCTT TAGCATCGTC GTTGGCTGAA CTACTTGTCA CTGAATCAGC

L.hes_c AGTACTGCTT TAGCATCGTC GTTGGCTGAA CTACTTGTCA CTGAATCAGC

L.geo_a AGTACTGCTT TAGCATCATC GTTGGCTGAA CTACTTGTCA CTGAATCAGC

S.gro_a AGTGCTGCCT TAGCGTCGTC CATGGCTGAA TTACTTGTCA CTGAGTCAGC

A.ape_a AGCACAGCTT TAGCTTCCTC TCTCGCGGAA ATTGTAATTA CAGAATCAGG

S.mim AGTACAGCAC TAGCATCATC ATTAGCAGAT CTCATAATAT CGGAAAATAA

**A.arg_TuSp1** TGAGAGCAAC TACAATAATC AGCTTTCTGA ACTAACAGGA ATACTTTCCA 400

**A.arg_TuSp1ψ** AGAGAGCAAC TACAATAATC AGCTTTCTGA ACTAACAGGA ATACTTTCCA

A.arg_a TGAGAGCAAC TACAATAATC AGCTTTCTGA ACTAACAGGA ATACTTTCCA

A.bru_a AGAAAGCAAT TACAGCAATC AGTTGTCTGA ACTAACAGGA ATTCTCTCCG

A.bru_b* AAAAAGCAAT TACAGCAATC AGCTGTCTGA ACTAACAGGA ATCCTCTCCG

N.ant_a TGGAAGCAGC TACCAAACTC AAATATCTGC CCTCACTAAT ATCCTATCCG

N.clava_a TGGAAGCAGC TACCAAACTC AAATATCTGC CCTCACTAAT ATCCTATCCG

P.tep_a TGGAATAGGC ATAAGCAAAC AGCTGTTTTC TCTGACGAAT ATACTTTCAC

P.tep_b TGGAATAGGC ATAAGCAAAC AGCTGTTTTC TCTGACGAAT ATACTTTCAC

P.tep_c TGGAATAGGC ATAAGCAAAC AGCTGTTTTC TCTGACGAAT ATACTTTCAC

L.hes_a AGAAGAAGAT ATTGATAATC AGGTAGTAGC TTTGTCAACA ATTCTTTCTC

L.hes_b AGAAGAAGAT ATTGATAATC AGGTAGTAGC TTTGTCAACA ATTCTTTCTC

L.hes_c TGAAGAAGAT ATTGATAATC AGGTAGTAGC TTTGTCAACA ATTCTTTCTC

L.geo_a AGAAGAAGAC ATTGAGAATC AGGTCACAGC TTTATCAGGA ATTCTTTCAC

S.gro_a AGAAACTGAG ATTGAGGAGC AAGTGTCAGC TCTGTCAGGA ATTCTTTCCC

A.ape_a GGGGCAGGAT TATTCTAAAC AAATAACAGA TTTAAATGGA ATCCTATCAA

S.mim TAACCAGGAA TATGCGAAAC AGATTTCTGC TTTATCGCAA ATACTTTCCA

**A.arg_TuSp1** ACTGTTTTAT CCAAACCACT GGATCGGATA ATCCAGCATT TGTATCCAGA 450

**A.arg_TuSp1ψ** ACTGTTTTAT CCAAACCACT GGATCGGATA ATCCAGCATT TGTATCCAGA

A.arg_a ACTGTTTTAT CCAAACCACT GGATCGGATA ATCCAGCATT TGTATCCAGA

A.bru_a ACTGTTTTAT CCAAACTACT GGATCGGACA ACCCAGCATT TGTGTCCAGA

A.bru_b* ACTGTTTTAT CCAAACTACT GGATCGGACA ATCCAGCATT TGTGTCCAGA

N.ant_a ATTGTTTTGT CACAACAACT GGATCAAACA ATCCTGCATT TGTATCAAGA

N.clava_a ATTGTTTTGT CACAACAACT GGATCAAACA ATCCTGCATT TGTATCAAGA

P.tep_a AATGTTTCAC TCAAACTACA GGATTACAAA GTCCAGAATT CGTGGATAAT

P.tep_b AATGTTTCAC TCAAACTACA GGATTACAAA GTCCAGAATT CGTGGATAAT

P.tep_c AATGTTTCAC TCAAACTACA GGATTACAAA GTCCAGAATT CGTGGATAAT

L.hes_a AGTGTTTCGT AGAAACCACA GGATCTCCCA ACCCAGCATT TGTAGCAAGT

L.hes_b AGTGTTTCGT AGAAACCACA GGATCTCCCA ACCCAGCATT TGTAGCAAGT

L.hes_c AGTGTTTCGT AGAAACCACA GGATCTCCCA ACCCAGCGTT TGTAGCAAGT

L.geo_a AGTGTTTTGT AGAAACCACA GGAGCTCCCA ACCCAGCATT TGTAGCAAGT

S.gro_a AATGTTTCAT TCAGATCACG GGAGCTCCTA ATCCAGTATT TGTATCGAAT

A.ape_a ACTGCTTTAT CCAAACAACA GGAGTAGAAA ACAAAAGATT CGTAAACAGC

S.mim AATGTTTCGT CCAAACAACG GGAAATTCCA ATACTCAATT CGTATCACGT

**A.arg_TuSp1** ATTCAGTCGC TCATATCAGT GCTTTCTCAG AATACTGATG TAAATATA 498

**A.arg_TuSp1ψ** ATTCAGTAGC -CATAACAGT GCTTTCTCAG AATACTGATG TAAATATA

A.arg_a ATTCAGTCGC TCATATCAGT GCTTTCTCAG AATACTGATG TAAATATA

A.bru_a ATTCAATCTC TCATTTCAGT GCTTTCCCAG AATGCAGATA CAAATATA

A.bru_b* ATTCAATCTC TCATTTCAGT GCTCTCCCAG AATGCAGATG TAAATATA

N.ant_a GTTCAAACAC TTATAGCAGT GCTTTCTCAA AGCAGCAGTA ATGCAATT

N.clava_a GTTCAAACAC TTATAGGAGT GCTTTCTCAA AGCAGCAGTA ATGCAATT

P.tep_a TTTCAAACAC TTCTTCAAAG AATCTCTGGA GTTGATATTG GAACTTCT

P.tep_b TTTCAAACAC TTCTTCAAAG AATCTCTGGA GTTGATATTG GAACTTCT

P.tep_c TTTCAAACAC TTCTTCAAAG AATCTCTGGA GTTGATATTG GAACTTCT

L.hes_a GTAAAATCGC TACTTGGAGT ATTATCACAG TCTGCAAGCA ATTATGAA

L.hes_b GTAAAATCGC TACTTGGAGT ATTATCACAG TCTGCAAGCA ATTATGAA

L.hes_c GTAAAATCGC TACTTGGAGT ATTATCACAG TCTGCAAGCA ATTATGAA

L.geo_a GTAAAATCAT TACTTGGAGT ATTATCAGAG GCTGCAAGCA ATGGTGAA

S.gro_a GTGAAATCTC TACTTGGTGT ACTATCACAG AGTTCAAGCA GTGCACAA

A.ape_a ATACAAAATC TCATAAGACT GCTTGCAGAA AGCGCAGTCT CAGAAACA

S.mim ATCAACAGAT TAGTAAGCGT CCTAGCTAAC GAAGCAACGA ATATTCAG

Figure 3. Nucleotide alignment of C-terminal encoding regions from the translated *A. argentata TuSp1* and *TuSp1*ψ characterized from BAC clones (bold) and previously characterized tubuliform spidroins. Complete sequence names and GenBank accession numbers are in Table S2.

**A.arg_TuSp1** GGCTTAGGTT CCTCTGCTGC CTCTGCCAGA GTGAGTAGTT TAGCCAATTC 50

**A.arg_TuSp1ψ** GGCTTAGGTT CCTCTGCTGC CTCTGACAGA GTGAGTAGTT TAGCCAATTC

A.arg_b GGCTTAGGTT CCTCTGCTGC CTCTGCCAGA GTGAGTAGTT TAGCCAATTC

A.arg_c GGCTTAGGTT CCTCTGCTGC CTCTGCCAGA GTGAGTAGTT TAGCCAATTC

A.aur_b GGCTTAGGCT CATCCGCTGC CACTGCCAGG GTGAGGAGTT TAGCCAACTC

A.aur_a GGCTTAGGCT CATCCGCTGC CACTGCCAGG GTGAGGAGTT TAGCCAACTC

A.bru_a GGCTTAGGCT CATCTGCTGC CACTGCCAGG GTGAGTAGTT TAGCCAACTC

A.bru_b GGCTTAGGTT CATCTGCTGC CACTGCCAGG GTGAGTAGTT TAGCCAACTC

G.hep GGCTTAGGTT CATCTGCTGC CTCAGCCAGG GTTGGTAGTT TAGCCAGCTC

C.mol GGCTTAGCAT CACCTGATGC CACTGCCAGA GTCAGTAGTT TATCTAATTC

A.gem GGTTTGGCCT CATCTGCTGC CAGTGCCAGA GTGAGTAGCT TAGCCCAATC

N.clava_b GGCTTATCAT CAGCGAGTGC GAGTGCAAGA GTTGGCAGTT TAGCTCAATC

N.ant_b GGCTTATCAT CAGCGAGTGC GAGTGCAAGA GTTAGCAGTT TAGCTCAATC

N.cla GGCTTATCAT CAGCGAGTGC TAATGCTAGA GTTAGCAGTT TAGCTCAATC

N.cru_a GGCTTAGCAT CATCCAGTGC GACTTCTAGA GTTGGCAGTT TAGCTCAATC

N.cru_b GGCTTAGCAT CATCCAGTGC GACTTCTAGA GTTGGCAGTT TAGCTCAATC

P.tep_c GGACTCGGAT CTGCGAGCGC AACTTCAAGA GTAAATAACA TTGAACAAAA

P.tep_d GGACTCGGAT CTGCGAGCGC AACTTCAAGA GTAAATAACA TTGAACAAAA

L.hes_f GGATTGGCTT CTACTGCAGC AACTTCAAGA ATTAATGACA TTGCACAAAG

L.hes_g GGATTGGCTT CTACTGCAGC AACTTCAAGA ATTAATGACA TTGCACAAAG

L.hes_d GGATTGGCTT CTACTGCAGC AACTTCAAGA ATTAATGACA TTGCACAAAG

L.hes_e GGATTGGCTT CTACTGCAGC AACTTCAAGA ATTAATGACA TTGCACAAAG

L.geo_c GGATTGGCTT CTACTGCAGC ATCTTCAAGA ATTAATGGTA TAGCGCAAAG

L.geo_b GGATTGGCTT CTACTGCAGC ATCTTCAAGA ATTAATGGTA TAGCGCAAAG

L.mac GGATTGGCTT CTACTGCAGC AACTTCAAGA ATTAATGACA TTGCACAAAG

L.tre GGATTGGCTT CTACTGCAGC AACTTTAAGA ATTAATGACA TTGCACAAGG

L.hass GGATTGGCTT CTACTGCAGC AACTTCAAGA ATTAATGACA TTGCACAAAG

S.gro GGGTTAGCTT CATCCGCAGC AACTTCGAGG ATCAATAGTG TAGCACAAGG

A.ape GGACTTTCTT CTGCTTCTGC ATCTTCTCGT GTAAATAGTT TAGCTTCATC

U.div GGTTTGTCTT CGTCTTCAGC CTCTTCACGA ATTAATAGCA TTGCTTCCGG

D.spi GGGCTGAGCT CAGCTGCTGC TACATCGCGT GCTAGTAGTC TGGCTTCTTC

S.mim CATCATTCCG CTGTTGGTTC TTCCTCTCAT ACTTCGTCTT TAGCTTCTTC

**A.arg_TuSp1** CGTTGCTTCT GCTATTTCTT CATCTGGAGG CTCC------ CTCAGTGTTC 100

**A.arg_TuSp1ψ** CCTTGCTTCC GCAATTTCTT CATCTGGAGG CTCC------ CTCAGCGTTC

A.arg_b CGTTGCTTCT GCTATTTCTT CATCTGGAGG CTCC------ CTCAGTGTTC

A.arg_c CGTTGCTTCT GCTATTTCTT CATCTGGAGG CTCC------ CTCAGTGTTC

A.aur_b CATTGCTTCT GCGATTTCTT CATCTGGAGG TTCC------ CTCAGTGTTC

A.aur_a CATTGCTTCT GCGATTTCTT CATCTGGAGG TTCC------ CTCAGTGTTC

A.bru_a CTTTGCTTCT GCGATTTCTT CATCTGGAGG TTCC------ CTCAGTGTTC

A.bru_b CATTGCTTCT GCGATTTCTT CATCTGGAGG TTCC------ CTCAGTGTTC

G.hep CATTGCTTCA GCTATTTCTT CATCCGGAGG TTTC------ CTCAGTGTTC

C.mol CATAGCTTCA GCAATTTCTT CATCCGGAGG TTCT------ TTAAATGTTC

A.gem AATTGCTTCT GCGATTTCTT CATCCGGTGG TACC------ TTGAGTGTTC

N.clava_b TCTGGCGTCT GCATTGTCGA CTTCTCGAGG TACT------ TTGAGTTTAT

N.ant_b TCTTGCGTCT GCATTGTCGA CTTCTCGAGG TACT------ TTGAGTTTAT

N.cla GTTTGCGTCT GCATTGTCGG CTTCTCGAGG TACT------ TTGAGTGTAT

N.cru_a TTTGGCATCC GCATTGCAAT CTTCGGGAGG TACA------ CTGGATGTTT

N.cru_b TTTGGCATCC GCATTGCAAT CTTCGGGAGG TACA------ CTGGATGTTT

P.tep_c CTTAGCTTCT GTCATAAAAT CCTCTGGATC TACAGCT--- CTGGACATAA

P.tep_d CTTAGCTTCT GTCATAAAAT CCTCTGGATC TACAGCT--- CTGGACATAA

L.hes_f TTTATCTTCA ACTCTA---T CTTCAGGATC GCAA------ TTAGCTCCAG

L.hes_g TTTATCTTCA ACTCTA---T CTTCAGGATC GCAA------ TTAGCTCCAG

L.hes_d TTTATCTTCA ACTCTA---T CTTCAGGATC GCAA------ TTAGCTCCAG

L.hes_e TTTATCTTCA ACTCTA---T CTTCAGGATC GCAA------ TTAGCTCCAG

L.geo_c TTTATCATCC ACCTTA---T CTTCAGGAAC ACAA------ TTAGCTCCAG

L.geo_b TTTATCATCC ACCTTA---T CTTCAGGAAC ACAA------ TTAGCTCCAG

L.mac TTTATCTTCA ACTCTA---T CTTCAGGATC GCAA------ TTAGCTCCAG

L.tre TTTATCTTCA ACTATA---T CTTCAGGATC GCAG------ TTAACTCCAG

L.hass TTTATCTTCA ACTATA---T CTTCAGGATC CCAA------ TTAGCTCCAG

S.gro CTTATCTTCT ACTATT---T CAGCAGGATC ACAA------ TTAGCTCCTG

A.ape AGTAGCTTCT GCTATA---G CCTCTGGTCA AGCT------ CTAAGTGCGG

U.div TTTATCGACT GCTCTATCAT CATCAAGAGG T--------- GTCAGCCTTG

D.spi TGTGGCGTCA GCCATTTCTT CAGCTGGAAG TGCTGGAGGT GTAGACGTAG

S.mim TTTGGTGTCT GCATTAACAT CGTCTGGAAG CTCTGGT--- CTAGATATTT

**A.arg_TuSp1** CAACCTTCTT G---AATTTT CTTTCATCCG TTGGGGCTCA AGTTAGCAGT 150

**A.arg_TuSp1ψ** GAACCTTTTT G---AATTTT CTTTCAAACG TTGGGGCTCA AGTTAGCAGT

A.arg_b CAACCTTCTT G---AATTTT CTTTCATCCG TTGGGGCTCA AGTTAGCAGT

A.arg_c CAACCTTCTT G---AATTTT CTTTCATCCG TTGGGGCTCA AGTTAGCAGT

A.aur_b CAGCCTTCTT G---AATCTC CTCTCATCCG TCGGGGCTCA AGTTAGTAGT

A.aur_a CAGCCTTCTT G---AATCTC CTCTCATCCG TCGGGGCTCA AGTTAGTAGT

A.bru_a CAACCTTCTT G---AATCTT CTTTCATCCG TTGGGGCCCA AGTTAGTAGT

A.bru_b CAACCTTCTT G---AATCTT CTTTCATCCA TTGGGGCGCA AGTTAGTAGT

G.hep CAACCTTCTT A---ACGCTT CTCTCATCAG TTGGGTCCCA AGTCGCTAGC

C.mol CTACTTTTTT G---AATCTG CTCTCATCAG TTGGATCCCA AATTAGTGGC

A.gem CTATCTTCTT G---AATCTT CTCTCATCCG CTGGAGCACA AGCTACTGCT

N.clava_b CAACCTTCTT G---AATCTC CTCTCTCCGA TTTCGTCAGA AATTCGAGCC

N.ant_b CAACCTTCTT G---AATCTC CTCTCTTCAA TTTCGTCAGA AATTCGAGCC

N.cla CAACCTTCTT A---ACTCTT CTCTCCCCGA TTTCGTCACA AATTCGAGCC

N.cru_a CGACCTTCTT G---AATCTT CTGTCTCCCA TTTCTACACA AATTCAAGCC

N.cru_b CGACCTTCTT G---AATCTT CTGTCTCCCA TTTCTACACA AATTCAAGCC

P.tep_c GGGATGCTCT T---CAAGAC GTTGACTATA TTTCATCTTT GATACAGAGT

P.tep_d GGGATGCTCT T---CAAGAC GTTGACTATA TTTCATCTTT GATACAGAGT

L.hes_f ATAATGTACT T---CCTGGT CTCATTCAAC TGTCTTCATC CATTCAAAGC

L.hes_g ATAATGTACT T---CCTGGT CTCATTCAAC TGTCTTCATC CATTCAAAGC

L.hes_d ATAATGTACT T---CCTGGT CTCATTCAAC TGTCTTCATC CATTCAAAGC

L.hes_e ATAATGTACT T---CCGGCT CTCATTCAAC TGTCTTCATC CATTCAAAGC

L.geo_c ATAATGTACT T---TCAGGT CTCTTCCAAC TGTCTTCTGC CATTCAAAGT

L.geo_b ATAATGTACT T---TCAGGT CTCTTCCAAC TGTCTTCTGC CATTCAAAGT

L.mac ATAATGTTCT T---CCGGGT CTCATTCAAC TGTCTTCATC CATTCAAAGC

L.tre ATAATGTAAT T---CCAGGT CTTATCCAAC TGTCTTCATC CATTCAAAGC

L.hass ATAATGTACT T---CCGGGT CTCTTCCAAC TGTCTTCATC TATTCAAAGC

S.gro ATTATGTACT T---CAAGGT TTGTTCCAAC TATCTTCGGC AATTCAAAAC

A.ape ATAGTTTTGC AAAATCGTTA TTGATCCAA- --GCTTCCCA AATTCAAAGT

U.div AAAACCTTTC G---AGTAGT TTATCTTCTG TGTTCTCAGA AATTCAGAAC

D.spi GATTATTCGC G---AGTGGC TTGTCTTCTC TCGTTTCTCA GATTCAAAGC

S.mim CTTCCTTTGC G---GACAAT TTGTCTTCTA TAGCATCTGA AATTCAAAGA

**A.arg_TuSp1** AGTAGTTCT- --TTGAATTC CTCC---GAA GTTACAAACG AAGTGTTACT 200

**A.arg_TuSp1ψ** AGTAGTTCT- --TTGAGTTC CTCG---GCA GTTACAACCC AAGTTTTACT

A.arg_b AGTAGTTCT- --TTGAATTC CTCC---GAA GTTACAAACG AAGTGTTACT

A.arg_c AGTAGTTCT- --TTGAATTC CTCC---GAA GTTACAAACG AAGTGTTACT

A.aur_b AGCAGCTCT- --TTGAATTC CTCG---GAA GTTACAAATG AAGTTTTACT

A.aur_a AGCAGCTCT- --TTGAATTC CTCG---GAA GTTACAAATG AAGTTTTACT

A.bru_a AGCAGTTCT- --TTGAGTTC CTTG---GAA GTTACAAACG AAGTTTTACT

A.bru_b AGCAGTTCT- --TTGAGTTC TTCCTCGGAA GTTACAACCC AAGTTTTACT

G.hep AGCAGCTCT- --TTGAGTTC CTCT---GAA GTAACAAACG AAGTTTTACT

C.mol AGTAGTTCC- --CTGAGTTC TTCT---CAG ATAAGGAGCC AAATTTTATT

A.gem AGCAGTTCT- --TTGAGTTC CTCG---CAA GTTACTAGCC AAGTTTTGCT

N.clava_b AATACTTCT- --CTTGATGG AACG---CAG GCGACTGTTG AAGCTTTACT

N.ant_b AGTACTTCT- --CTTGATGG AACG---CAG GCGACTGTTG AAGTTTTACT

N.cla AATACTTCT- --CTTGATGG AACA---CAA GCTACTGTTC AAGTTTTACT

N.cru_a AATACTTCT- --CTAAATGC ATCA---CAG GCGATTGTCC AAGTTTTACT

N.cru_b AATACTTCT- --CTAAATGC ATCA---CAG GCGATTGTCC AAGTTTTACT

P.tep_c GAGAGACCGG AATTGAGTTC CTCG---CAA GTTTTGACTG AATCTTTGCT

P.tep_d GAGAGACCGG AATTGAGTTC CTCG---CAA GTTTTGACTG AATCTTTGCT

L.hes_f GGAAATCCTG ATTTAGACCC TGCT---GGT GTTTTGATCG AGTCATTATT

L.hes_g GGAAATCCTG ATTTAGACCC TGCT---GGT GTTTTGATCG AGTCATTATT

L.hes_d GGAAATCCTG ATTTAGACCC TGCT---GGT GTTTTGATCG AGTCATTATT

L.hes_e GGAAATCCTG ATTTAGACCC TGCT---GGT GTTTTGATCG AGTCATTATT

L.geo_c GGAAATCCTG AATTAGACAC TTCT---GGT GTTCTGATAG AATCGTTATT

L.geo_b GGAAATCCTG AATTAGACAC TTCT---GGT GTTCTGATAG AATCGTTATT

L.mac GGAAATCCTG ATTTAGACCC TGCT---GGT GTTTTGATCG AGTCATTATT

L.tre GGAAATCCTG ATTTAGACTC TGCT---GGT GTTTTGATCG AATCATTATT

L.hass GGAAATCCTG GTTTAGATTC TGCT---GGT GTTTTGATCG AATCATTATT

S.gro TTGAATCCTC AGTTAGACTC TTCA---ACT ATATTGATTG AATCTTTATT

A.ape AGTGCACCTA GT---TTTAA AGCAGATGAT GTAGTCCACG AATCTCTCCT

U.div AATAGTTTCG GGGTTTCTGC TGAG---CAA GCTTTGATTC AAGCTCTGTT

D.spi AGCAATCTTG GATTGCAGCC GGAT---CAA GTTTTGCTAG AAGCTCTACT

S.mim AACTCACCTG GATTAGAATC AAAG---GAA GTATTAGTCG AAACCCTCTT

**A.arg_TuSp1** TGAAGCTATC GCGGCACTCT TGCAAGTCCT CAACGGAGCT CAAATAACAT 250

**A.arg_TuSp1ψ** TGAAGCCATT GCGGCTCTCT TGCAAGTCAT TAATGGGGCT CGAATAACTT

A.arg_b TGAAGCTATC GCGGCACTCT TGCAAGTCCT CAACGGAGCT CAAATAACAT

A.arg_c TGAAGCTATC GCGGCACTCT TGCAAGTCCT CAACGGAGCT CAAATAACAT

A.aur_b TGAAGCTATA GCGGCGCTCT TGCAAGTTAT CAACGGAGGT TCAATAACAT

A.aur_a TGAAGCTATA GCGGCGCTCT TGCAAGTTAT CAACGGAGGT TCAATAACAT

A.bru_a TGAAGCTATT GCGGCTCTCT TGCAAGTTAT CAACGGAGGT TCAATAACAT

A.bru_b TGAAGCTATT GCGGCGCTCT TGCAAGTTAT CAACGGGGCT CAAATAACTT

G.hep TGAAACTATT TCGGCGCTTT TGCAAGTTAT CAATGGAGCT GGAATA----

C.mol GGAGGGTATT GCTGCCCTTT TGCAAGTAAT CAATGGAGCC AAAATATCTT

A.gem GGAAGGTATT GCAGCTCTCT TGCAAGTTAT CAACGGAGCT CAGATTAGAT

N.clava_b GGAAGCTTTA GCTGCTCTCC TGCAAGTTAT CAATGGAGCA CAGATAACCG

N.ant_b GGAAGCTTTA GCTGCTCTCC TGCAAGTTAT CAATGGAGCA CAGATAACAG

N.cla CGAAGCTTTA GCTGCTCTCC TACAAGTTAT CAATGCAGCG CAGATAACAG

N.cru_a TGAAGCTGTA GCTGCTCTGC TGCAAATTAT CAACGGAGCT CAAATAACTT

N.cru_b TGAAGCTGTA GCTGCTCTGC TGCAAATTAT CAACGGAGCT CAAATAACTT

P.tep_c CGAATACACG TCCGCACTTT TACAACTTCT TCAAAAATCA ACCATTACAA

P.tep_d CGAATACACG TCCGCACTTT TACAACTTCT TCAAAAATCA ACCATTACAA

L.hes_f AGAATACACT TCCGCACTTT TAGCTCTTCT TCAAAACGCT CAAATTACAA

L.hes_g AGAATACACT TCCGCACTTT TAGCTCTTCT TCAAAACGCT CAAATTACAA

L.hes_d AGAATACACT TCCGCACTTT TAGCTCTTCT TCAAAACGCT CAAATTACAA

L.hes_e AGAATACACT TCCGCACTTT TAGCTCTTCT TCAAAATGCT CAAATTACAA

L.geo_c TGAATACACT TCCGCACTTT TAGCTCTCCT TCAGAATGCA CAAGTTACAA

L.geo_b TGAATACACT TCCGCACTTT TAGCTCTCCT TCAGAATGCA CAAGTTACA-

L.mac AGAATACACT TCCGCACTTT TAGCTCTTCT TCAAAATGCT CAAATTACA-

L.tre TGAATACACT TCCGCACTTT TAGCTCTTCT TCAAAATGCT CAAATTACA-

L.hass TGAATACACT TCCGCACTTT TAGCTCTTCT TCAGAATGCT CAGATTACA-

S.gro TGAATATACT TCTGCTCTGC TAGCTCTTCT TCAAAATGCA CATATAGAGG

A.ape TGAAGGAATT TCAGCTCTTA TCCAAGTTAT CAACAGCAGT TATGGTTCAC

U.div TGAAGTCTTA ACTGGTACAG TGCAAGTTCT TAACAGAGGT CAAACATCGT

D.spi TGAAGGATAT TCAGCTTTAG CCCAAGTATT AATTAGCTCT CAGATTTCAT

S.mim AGATGCTGTA TCGGCGCTTT ACCAAATTCT CTCAAATGCC CAGATTTCTT

**A.arg_TuSp1** CAGTTAATCT GAGAAACGTT CCGAATGCTC AACAGGCTTT GGTGCAAGCA 300

**A.arg_TuSp1ψ** CAGTTAATCT TTCAAATTTT TCGAATGTCA ATCGAGAGTT TGTTGATTCT

A.arg_b CAGTTAATCT GAGAAACGTT CCGAATGCTC AACAGGCTTT GGTGCAAGCA

A.arg_c CAGTTAATCT GAGAAACGTT ---------- ---------- ----------

A.aur_b CAGTTGATCT TAGAAACGTT ---------- ---------- ----------

A.aur_a CAGTTGATCT TAGAAACGTT CCGAATGCTC AGCAGGATTT GGTGAATGCT

A.bru_a CAGTTGATCT TAGATACGTT CCGAATGCTC AGCAGGATTT GGTGAACGCT

A.bru_b CAGTTAATTT TTCAAATGTT TCGAATGTAA ACCGAGCACT TGTAGATTCT

G.hep ---------- ---------- ---------- ---------- ----------

C.mol CGGTTAATCT TGCAAACGTT ---------- ---------- ----------

A.gem CAGTTAATCT TGCAAACGTT CCCAATGTTC AACAGGCATT GGTGAGTGCT

N.clava_b ATGTCAATGT TTCTAGCGTC CCCAGCGTGA ATGCAGCCCT GGCTTCTGCT

N.ant_b ACGTCAATGT TTCTAGCGTC CCCAGTGTGA ATGCAGCTCT GGTGTCTGCT

N.cla AAGTCAATGT TTCAAACGTC TCCAGCGCAA ACGCAGCTCT GGTGTCTGCA

N.cru_a CTGTCAATTT TGGCAGTGTC TCCAGCGTAA ACACAGCCTT GGCAACTGCT

N.cru_b CTGTCAATTT TGGCAGTGTC TCCAGCGTAA ACACAGCCTT GGCAACTGCT

P.tep_c ATGTGAACGT ATCGAATGGA CCTCCAGTGA ATGTAGAGCT AGCGAGATAT

P.tep_d ATGTGAACGT ATCGAATGGA CCTCCAGTGA ATGTAGAGCT AGCGAGATAT

L.hes_f CTTATGATGC TGCGACTTTA CCTGCATTCA GTACAGCTCT TGTAAATTAC

L.hes_g CTTATGATGC TGCGACTTTA CCTGCATTCA GTACAGCTCT TGTAAATTAC

L.hes_d CTTATGATGC TGCGACTTTA CCTGCATTCA ATACAGCTCT TGTAAATTAC

L.hes_e CTTTTGATGC TGCGACTTTA CCTGCATTCA ATACAGCTCT TGTAAATTAC

L.geo_c CTTTTGATGT TGCGACTTTT CCTGCATTCA ATACAGCTCT TGTAAATTAC

L.geo_b ---------- ---------- ---------- ---------- ----------

L.mac ---------- ---------- ---------- ---------- ----------

L.tre ---------- ---------- ---------- ---------- ----------

L.hass ---------- ---------- ---------- ---------- ----------

S.gro CCATAAATAT TTCAGCTTTA CCTCCCGTGA ATACAGCTCT TGTAAATTAC

A.ape CTTTGAGTTT GTCCAATGCC CAGACTGTTA ACGCTGGACT TGTAAACTAT

U.div TCGTGAGTGT ATCATCTCCA ACTGTGATTA GCAGTTCTTT C---------

D.spi CTGTAAGTGT ATCATCATCT TCGGCATTGG GGCCAGCTCT CTTAAACTAC

S.mim CGGTTAATAT TCCAGCTTCA TTTGATGTTA AGAGCGCGCT TGCTAATGCC

**A.arg_TuSp1** TTGTCTGGT- -------- 318

**A.arg_TuSp1ψ** CTTGTAAGT- --------

A.arg_b TTGTCTGGT- --------

A.arg_c ---------- --------

A.aur_b ---------- --------

A.aur_a CTATCTGGT- --------

A.bru_a TTATCTGGT- --------

A.bru_b CTTGTAGGTT CATTTGCT

G.hep ---------- --------

C.mol ---------- --------

A.gem CTTTCTGGT- --------

N.clava_b CTTGTTGCT- --------

N.ant_b CTTGTTGCT- --------

N.cla CTTGCTGGT- --------

N.cru_a CTCGCTGGT- --------

N.cru_b CTCGCTGGT- --------

P.tep_c CTTTCTCAAT CT------

P.tep_d CTTTCTCAAT CT------

L.hes_f CTTGTTCCCC TTGTT---

L.hes_g CTTGTTCCCC TTGTT---

L.hes_d CTTGTTCCCC TTGTT---

L.hes_e CTTGTTCCCC TTGTT---

L.geo_c CTGGTTCCCC TTGTT---

L.geo_b ---------- --------

L.mac ---------- --------

L.tre ---------- --------

L.hass ---------- --------

S.gro CTAATTCCAC TTATT---

A.ape TTCCTTGTT- --------

U.div ---------- --------

D.spi CTTGTAGGT- --------

S.mim CTTTCTGCC- --------

Figure S4. Nucleotide alignment of 5’ region PCR amplified variants (numbered) from *A. argentata, A. aurantia,* and *A. trifasciata* with downloaded *Argiope TuSp1* sequences (lettered). A.bru_b* sequence includes corrections reported by Han and Nakagaki (2013). Alignment gaps indicated by dashes. GenBank accession numbers and full names for variants and downloaded sequences in Tables S2, S3.

**A.arg_TuSp1** TCAAGTGATA CTCAATGCCG TTTCAACAAA TACTGGCGCC ACAGCATCAG 50

**A.arg_TuSp1ψ** CCAAGTGATA CTCAATGCCG TTTCAAGCAA TACTGGCCCC ACAGCATCAG

A.arg_a TCAAGTGATA CTCAATGCCG TTTCAACAAA TACTGGCGCC ACAGCATCAG

A.arg_v2 TCAAGTGATA CTCAATGCCG TTTCAACAAA TACTGGCGCC ACAGCATCAG

A.arg_v3 CCAAGTGATA CTCAATGCCG TTTCAAGCAA TACTGGCCCC ACAGCATCAG

A.arg_v4 CCAAGTGATA CTCAATGCCG TTTCAAGCAA TACTGGCCCC ACAGCATCAG

A.bru_a TCAGGTGATA CTCAATGCTG TTTCAAGCAA CACTGGCGCT ACAGCATCGG

A.bru_b* CCAGGTGATA CTCAATGCCG TTTCAAGCAA CACTGGCGCC ACAGCATCGG

A.tri_v1 CCAGGTGATA CTGAATGCCG TCTCCAGCAA CACTGGCGCC ACAGCATCGG

A.tri_v2 CCAGGTGATA CTGAATGCCG TCTCCAGCAA CACTGGCGCC ACAGCATCGG

A.tri_v3 CCAGGTGATA CTGAATGCCG TCTCCAGCAA CACTGGCGCC ACAGCATCGG

A.tri_v4 CCAGGTGATA CTGAATGCCG TCTCCAGCAA CACTGGCGCC ACAGCATCGG

A.aur_v1 TCAGGTGATA CTCAATGCTG TTTCAAGCAA CAATGGCGCC ACAGCATCGG

A.aur_v2 TCAGGTGATA CTCAATGCTG TTTCAAGCAA CAATGGCGCC ACAGCATCGG

A.aur_v3 TCAGGTGATA CTCAATGCTG TTTCAAGCAA CAATGGCGCC ACAGCATCGG

A.aur_v4 CCAAGTGATA CTCAATGCCG TTTCAAGCAA CACTGGCGCC ACAACATCGG

**A.arg_TuSp1** CCAGAGCTCA AGCTTTAAGT ACAGCGCTCG CATCCTCTCT CACAGATCTC 100

**A.arg_TuSp1ψ** CCAGAGCTCA AGCTTTAAGT ACAGCGCTCG CATCTTCTCT CACAGATCTG

A.arg_a CCAGAGCTCA AGCTTTAAGT ACAGCGCTCG CATCCTCTCT CACAGATCTC

A.arg_v2 CCAGAGCTCA AGCTTTAAGT ACAGCGCTCG CATCCTCTCT CACAGATCTC

A.arg_v3 CCAGAGCTCA AGCTTTAAGT ACAGCGCTCG CATCTTCTCT CACAGATCTG

A.arg_v4 CCAGAGCTCA AGCTTTAAGT ACAGCGCTCG CATCTTCTCT CACAGATCTG

A.bru_a CCAGAGCTCA AGCTTTAAGT ACAGCGCTTG CATCTTCTCT GACAGATCTG

A.bru_b* CCAGAGCTCA AGCTTTAAGT ACAGCGCTTG CATCTTCTCT GACAGATCTG

A.tri_v1 CCAGAGCCCA AGCGTTAAGC ACTGCGCTCG CATCTTCTCT GACGGATCTG

A.tri_v2 CCAGAGCCCA AGCGTTAAGC ACTGCGCTCG CATCTTCTCT GACGGATCTG

A.tri_v3 CCAGAGCCCA AGCGTTAAGC ACTGCGCTCG CATCTTCTCT GACGGATCTG

A.tri_v4 CCAGAGCCCA AGCGTTAAGC ACTGCGCTCG CATCTTCTCT GACGGATCTG

A.aur_v1 CCAGAGCTCA AGCTTTAAGT ACAGCGCTTG CATCTTCTCT GACAGATCTG

A.aur_v2 CCAGAGCTCA AGCTTTAAGT ACAGCGCTTG CATCTTCTCT GACAGATCTG

A.aur_v3 CCAGAGCTCA AGCTTTAAGT ACAGCGCTTG CATCTTCTCT GACAGATCTG

A.aur_v4 CCAGAGCTCA AGCTTTAAGT ACAGCGCTTG CATCTTCTCT GACAGATCTG

**A.arg_TuSp1** CTCATTGCAG AGTCGGCTGA GAGCAACTAC AATAATCAGC TTTCTGAACT 150

**A.arg_TuSp1ψ** CTCATTGCAG AGTCGGCAGA GAGCAACTAC AATAATCAGC TTTCTGAACT

A.arg_a CTCATTGCAG AGTCGGCTGA GAGCAACTAC AATAATCAGC TTTCTGAACT

A.arg_v2 CTCATTGCAG AGTCGGCTGA GAGCAACTAC AATAATCAGC TTTCTGAACT

A.arg_v3 CTCATTGCAG AGTCGGCAGA GAGCAACTAC AATAATCAGC TTTCTGAACT

A.arg_v4 CTCATTGCAG AGTCGGCAGA GAGCAACTAC AATAATCAGC TTTCTGAACT

A.bru_a CTCATTGCAG AGTCGGCAGA AAGCAATTAC AGCAATCAGT TGTCTGAACT

A.bru_b* CTCATTGCAG AGTCGGCAAA AAGCAATTAC AGCAATCAGC TGTCTGAACT

A.tri_v1 CTCATTGAAG AGTCTGCTGA AAGCAACTAC AACAATCAGC TCTCAGAACT

A.tri_v2 CTCATTGAAG AGTCTGCAGA AAGCAACTAC AACAATCAGC TCTCAGAACT

A.tri_v3 CTCATTCAAG AGTCTGCTGA AAGCAACTAC AACAATCAGC TCTCAGAACT

A.tri_v4 CTCATTGAAG AGTCTGCAGA AAGCAACTAC AACAATCAGC TCTCAGAACT

A.aur_v1 CTCATTGCAG AGTCGGCAGA AAGCAATTAC GGCAATCAGC TGTCAGAACT

A.aur_v2 CTCATTGCAG AGTCGGCAGA AAGCAATTAC GGCAATCAGC TGTCAGAACT

A.aur_v3 CTCATTGCAG AGTCGGCAGC AAGCAGTTAC AGCAATCAGC TGTCAGAACT

A.aur_v4 CTCATTGCAG AGTCGGCAGC AAGCAGTTAC AGCAATCAGC TGTCAGAACT

**A.arg_TuSp1** AACAGGAATA CTTTCCAACT GTTTTATCCA AACCACTGGA TCGGATAATC 200

**A.arg_TuSp1ψ** AACAGGAATA CTTTCCAACT GTTTTATCCA AACCACTGGA TCGGATAATC

A.arg_a AACAGGAATA CTTTCCAACT GTTTTATCCA AACCACTGGA TCGGATAATC

A.arg_v2 AACAGGAATA CTTTCCAACT GTTTTATCCA AACCACTGGA TCGGATAATC

A.arg_v3 AACAGGAATA CTTTCCAACT GTTTTATCCA AACCACTGGA TCGGATAATC

A.arg_v4 AACAGGAATA CTTTCCAACT GTTTTATCCA AACCACTGGA TCGGATAATC

A.bru_a AACAGGAATT CTCTCCGACT GTTTTATCCA AACTACTGGA TCGGACAACC

A.bru_b* AACAGGAATC CTCTCCGACT GTTTTATCCA AACTACTGGA TCGGACAATC

A.tri_v1 AAAAGAAATC CTTTCCAAGT GTTTTATCCA AACTACTGGA TCAGACAATC

A.tri_v2 AAAAGAAATC CTTTCCAAGT GTTTTATCCA AACTACTGGA TCAGACAATC

A.tri_v3 AAAAGAAATC CTTTCCAAGT GTTTTATCCA AAGTACTGGA ACAGACAATC

A.tri_v4 AAAAGAAATC CTTTCCAAGT GTTTTATCCA AACTACTGGA TCAGACAATC

A.aur_v1 AACAGGAATC CTCTCCAACT GTTTTATCCA AACTACTGGA TCGGACAATC

A.aur_v2 AACAGGAATC CTCTCCAACT GTTTTATCCA AACTACTGGA TCGGACAATC

A.aur_v3 AACAGGAATC CTCTCCGACT GTTTTATCCA AACTACTGGA TCGGACAATC

A.aur_v4 AACAGGAATC CTCTCCGACT GTTTTATCCA AACTACTGGA TCGGACAATC

**A.arg_TuSp1** CAGCATTTGT ATCCAGAATT CAGTCGCTCA TATCAGTGCT TTCTCAGAAT 250

**A.arg_TuSp1ψ** CAGCATTTGT ATCCAGAATT CAGTAGCCA- TAACAGTGCT TTCTCAGAAT

A.arg_a CAGCATTTGT ATCCAGAATT CAGTCGCTCA TATCAGTGCT TTCTCAGAAT

A.arg_v2 CAGCATTTGT ATCCAGAATT CAGTCGCTCA TATCAGTGCT TTCTCAGAAT

A.arg_v3 CAGCATTTGT ATCCAGAATT CAGTAGCCCA TAACAGTGCT TTCTCAGAAT

A.arg_v4 CAGCATTTGT ATCCAGAATT CAGTAGCCCA TAACAGTGCT TTCTCAGAAT

A.bru_a CAGCATTTGT GTCCAGAATT CAATCTCTCA TTTCAGTGCT TTCCCAGAAT

A.bru_b* CAGCATTTGT GTCCAGAATT CAATCTCTCA TTTCAGTGCT CTCCCAGAAT

A.tri_v1 CAGCTTTTGT GTCCAGAATT CAATCGCTCA TTTCAGTGCT TTCCCAGAAT

A.tri_v2 CAGCGTTTGT GTCCAGAATT CAATCGCTCA TTTCAGTGCT TTCCCAGAAT

A.tri_v3 CAGCTTTTGT GTCCAGAATT CAATCGCTCA TTTCAGTGCT TTCCCAGAAT

A.tri_v4 CAGCGTTTGT GTCCAGAATT CAATCGCTCA TTTCAGTGCT TTCCCAGAAT

A.aur_v1 CAGCATTTGT GTCTAGAATT CAATCTCTTA TTTCAGTGCT TTCCCAGAAT

A.aur_v2 CAGCATTTGT GTCTAGAATT CAATCTCTTA TTTCAGTGCT TTCCCAGAAT

A.aur_v3 CAGCATTTGT GTCTAGAATT CAATCTCTTA TTTCAGTGCT TTCCCAGAAT

A.aur_v4 CAGCATTTGT GTCTAGAATT CAATCTCTTA TTTCAGTGCT TTCCCAGAAT

**A.arg_TuSp1** ACTGATGTAA ATATAATTTC TACAGCGGGG CTACCAACAG CT------AT 300

**A.arg_TuSp1ψ** ACTGATGTAA ATATAATTTC CACAGCGGGA CTACCAACAG CT------AT

A.arg_a ACTGATGTAA ATATAATTTC TACAGCGGGG CTACCAACAG CT------AT

A.arg_v2 ACTGATGTAA ATATAATTTC TACAGCGGGG CTACCAACAG CT------AT

A.arg_v3 ACTGATGTAA ATATAATTTC CACAGCGGGA CTACCAACAG CT------AT

A.arg_v4 ACTGATGTAA ATATAATTTC CACAGCGGGA CTACCAACAG CT------AT

A.bru_a GCAGATACAA ATATAATTTC CTCAGCAGGG ATACCTTCAG TGTCAGGAAG

A.bru_b* GCAGATGTAA ATATAATTTC CTCGGCAGGG ATACCTTCAG TGTCAGGAAG

A.tri_v1 GCAGATGCAA ATATCGTTCT CACAGGAGGA CTACCTTCGG TGTCAGGAAT

A.tri_v2 GCAGATGCAA ATATCGTTCT CACAGGGGGA CTACCTTCGG TGTCAGGAAT

A.tri_v3 GCAGATGCAA ATATCGTTCT CACAGGGGGA CTACCTTCGG TGTCAGGAAT

A.tri_v4 GCAGATGCAA ATATCGTTCT CACAGGGGGA CTACCTTCGG TGTCAGGAAT

A.aur_v1 GCAGACGCAA ATATAATATC CTCAGGGGGG ATAGCTTCAG TTTCAGGAAG

A.aur_v2 GCAGACGCAA ATATAATATC CTCAGGGGGG ATAGCTTCAG TTTCAGGAAG

A.aur_v3 GCAGACGCAA ATATAATATC CTCAGGGGGG ATAGCTTCAG TGTCAGGAAG

A.aur_v4 GCAGACGCAA ATATAATATC CTCAGGGGGG ATAGCTTCAG TGTCAGGAAG

**A.arg_TuSp1** TAGCGGTGCT GGTGGATTCG GATTCGCTAA AACT------ ------GCAA 350

**A.arg_TuSp1ψ** TAGCGGTGCT GGTGGATTAG GATTCGCTAA AACT------ ------GCAA

A.arg_a TAGCGGTGCT GGTGGATTCG GATTCGCTAA AACT------ ------GCAA

A.arg_v2 TAGCGGTGCT GGTGGATTCG GATTCGCTAA AACT------ ------GCAA

A.arg_v3 TAGCGGTGCT GGTGGATTAG GATTCGCTAA AACT------ ------GCAA

A.arg_v4 TAGCGGTGCT GGTGGATTAG GATTCGCTAA AACT------ ------GCAA

A.bru_a AAGGGGAGCT GGTGGATTAG GATTCGATAA CACCGCAAGA CAGTCCGCAA

A.bru_b* AAGTGGAGCT AGAGGA---G GATTCTCTAA CGCCGCAAGC CAGTCCGCAA

A.tri_v1 TAGCGCAGCT GGAGCGCTTG GATTCGCAAA CGCAGAAAGC CAGTCCGCAA

A.tri_v2 TAGCGCAGCT GGAGCGCTTG GATTCGCAAA CGCAGAAAGC CAGTCCGCAA

A.tri_v3 TAGCGCAGTT GGAGCGCTTG GACTCGCAAA CGCAGCAAGC CAGTCCACAA

A.tri_v4 TAGCGCAGTT GGAGCGCTTG GACTCGCAAA CGCAGCAAGC CAGTCCGCAA

A.aur_v1 AACCGGAGCT GGTGGATTAA GATTTCCTAA CGCCGCAAGT CAGTCCGCAA

A.aur_v2 AACCGGAGCT GGTGGATTAA GATTTCCTAA CGCCGCAAGT CAGTCCGCAA

A.aur_v3 AACCGGAGCT GGTGGATTAA GATTTCCTAA CGCCGCAAGT CAGTCCGCAA

A.aur_v4 AACCGGAGCT GGTGGATTAA GATTTCCTAA CGCCGCAAGT CAGTCCGCAA

**A.arg_TuSp1** GCAGCAGTGC CTCGCAAGCA AGTGCCTCTT CCTTCGCACA AGCATCCTCA 400

**A.arg_TuSp1ψ** GCAGCAGTGC CTCGCAAGCA AGTGCCTCTT CCTTCGCACA AGCATCCTCA

A.arg_a GCAGCAGTGC CTCACAAGCA AGTGCCTCTT CCTTCGCACA AGCATCCTCA

A.arg_v2 GCAGCAGTGC CTCGCAAGCA AGTGCCTCTT CCTTCGCACA AGCATCCTCA

A.arg_v3 GCAGCAGTGC CTCGCAAGCA AGTGCCTCTT CCTTCGCACA AGCATCCTCA

A.arg_v4 GCAGCAGTGC CTCGCAAGCA AGTGCCTCTT CCTTCGCACA AGCATCCTCA

A.bru_a GCAGCGCTGC CTCGCAGGCA AGTGCATCTT CATTCGCACA AGCATCCTCT

A.bru_b* GCAGYGCTGC CTCGCAAGCA AGTGCCTCTT CATTCGCACA AGCATCCTCT

A.tri_v1 GCAGCAGTGC CTCGCAAGCA AGTGCCTCTT CATTCGCACA AGCCTCCTCT

A.tri_v2 GCAGCAGTGC CTCGCAAGCA AGTGCCTCTT CATTCGCACA AGCCTCCTCT

A.tri_v3 GCAGCAGTGC CTCGCAAGCA AGTGCCTCTT CATTCGCACA AGCCTCCTCT

A.tri_v4 GCAGCAGTGC CTCGCAAGCA AGTGCCTCTT CATTCGCACA AGCCTCCTCT

A.aur_v1 GCAGCGCTGC CTCGCAAGCA AGTGCCTCTT CATTCGCACA AGCATCCTCT

A.aur_v2 GCAGCGCTGC CTCGCAAGCA AGTGCCTCTT CATTCGCACA AGCATCCTCT

A.aur_v3 GCAGCGCTGC CTCGCAAGCA AGTGCCTCTT CATTCGCACA AGCATCCTCT

A.aur_v4 GCAGCGCTGC CTCGCAAGCA AGTGCCTCTT CATTCGCACA AGCATCCTCT

**A.arg_TuSp1** GCTTCCCTTG CAGCCTCAAG CTCTTTCTCC AGTGCCTTCT CTTCGGCCAA 450

**A.arg_TuSp1ψ** TCTTCCCTTG CAGCCTCAAA TTCTTTCTCA AGTGCCTTCT CTTCGGCCAA

A.arg_a GCTTCCCTGG CAGCCTCAAG CTCTTTCTCC AGTGCCTTCT CTTCGGCCAA

A.arg_v2 GCTTCCCTTG CAGCCTCAAG CTCTTTCTCC AGTGCCTTCT CTTCGGCCAA

A.arg_v3 GCTTCCCTTG CAGCCTCAAG CTCTTTCTCC AGTGCCTTCT CTTCGGCCAA

A.arg_v4 TCTTCCCTTG CAGCCTCAAA TTCTTTCTCA AGTGCCTTCT CTTCGGCCAA

A.bru_a GCTTCCCTTG CAGCCTCAAG CGCTTTCTCC AGTGCCTTCT CCTCGGCAAA

A.bru_b* GCTTCCCTTG CAGCCTCAAG TTCTTTCTCC AGTGCCTTCT CTTCGGCCAA

A.tri_v1 GCTTCTCTTG CAGCCTCAAG TTCTTTCTCC AGTGCCTTCT CTTCGGCCAC

A.tri_v2 GCTTCCCTTG CAGCCTCAAG TTCTTTCTCC AGTGCCTTCT CTTCGGCCAC

A.tri_v3 GCTTCCCTTG CAGCCTCAAG TTCTTTCTCC AGTGCCTTCT CTTCGGCCAC

A.tri_v4 GCTTCCCTTG CAGCCTCAAG TTCTTTCTCC AATGCCTTCT CTTCGGCCAC

A.aur_v1 GCTTCCCTTG CAGCGTCAAG TTCTTTCTCC AGTGCCTTCT CTTCGGCCAA

A.aur_v2 GCTTCCCTTG CAGCCTCAAG TTCTTTCTCC AGTGCCTTCT CTTCGGCCAA

A.aur_v3 GCTTCCCTTG CAGCCTCAAG TTCTTTCTCC AGTGCCTTCT CTTCGGCCAA

A.aur_v4 GCTTCCCTTG CAGCCTCAAG TTCTTTCTCC AGTGCCTTCT CTTCGGCCAA

**A.arg_TuSp1** TACCCTCTCA GCTCTCGGTA ACGTTGCTTA TCAACTAGGC TTCAACGTAG 500

**A.arg_TuSp1ψ** TACCCTCTCA GCTCTCGGAA ACGTTGCTTA TCAACTAGGC TTCAACGTAG

A.arg_a TACCCTCTCA GCTCTCGGTA ACGTTGCTTA TCAACTAGGC TTCAACGTAG

A.arg_v2 TACCCTCTCA GCTCTCGGTA ACGTTGCTTA TCAACTAGGC TTCAACGTAG

A.arg_v3 TACCCTCTCA GCTCTCGGTA ACGTTGCTTA TCAACTAGGC TTCAACGTAG

A.arg_v4 TACCCTCTCA GCTCTCGGAA ACGTTGCTTA TCAACTAGGC TTCAACGTAG

A.bru_a TTCCCTCTCA GCTCTCGGCA ACGTAGCTTA TCAATTAGGA TTCAACGTAG

A.bru_b* TTCCCTCTCA GCTCTCGGCA ACGTTGCTTA TCAATTAGGC TTCAACGTAG

A.tri_v1 TTCAGTCTCG GCTCTCGGCA ATGTCGCTTA TCAGTTGGGC TTCAACACAG

A.tri_v2 TTCCCTCTCG GCTCTCGGCA ACGTCGCTTA TCAGTTGGGC TTCAACACAG

A.tri_v3 TTCCCTCTCA GCTCTCGGCA ACGTCGCTTA TCAGTTGGGC TACAACGCAG

A.tri_v4 TTCCCTCTCA GCTCTCGGCA ACGTCGCTTA TCAGTTGGGC TACAACGCAG

A.aur_v1 TTCACTCTCA GCTCTCGGCA ACGTAGCTTA TCAATTAGGC TTCAACGTAG

A.aur_v2 TTCACTCTCA GCTCTCGGCA ACGTAGCTTA TCAATTAGGC TTCAACGTAG

A.aur_v3 TTCCCTCTCA GCTCTCGGCA ACGTTGCTTA TCAATTAGGC TTCAACGTAG

A.aur_v4 TTCCCTCTCA GCTCTCGGCA ACGTTGCTTA TCAATTAGGC TTCAACGTAG

**A.arg_TuSp1** CTAATACTCT CGGTCTCGGC AACGCCGCAG GCCTCGGTGC CGCCTTATCT 550

**A.arg_TuSp1ψ** CTAATACTCT CGGTCTCGGC AACGCCGCAG GCCTCGGTGC CGCCTTATCT

A.arg_a CTAATACTCT CGGTCTCGGC AACGCCGCAG GCCTCGGTGC CGCCTTATCT

A.arg_v2 CTAATACTCT CGGTCTCGGC AACGCCGCAG GCCTCGGTGC CGCCTTATCT

A.arg_v3 CTAATACTCT CGGTCTCGGC AACGCCGCAG GCCTCGGTGC CGCCTTATCT

A.arg_v4 CTAATACTCT CGGTCTCGGC AACGCCGCAG GCCTCGGTGC CGCCTTATCT

A.bru_a CTAATACTCT CGGTATCGGC AACGCTCCAG GCCTCGGTAA TGCCTTATCT

A.bru_b* CTAACACTCT CGGTATCGGC AACGCTCCAG GCCTCGGTGC TGCCTTATCT

A.tri_v1 CTACCAATCT CGGAATCGCC AATCCTGCAG GCCTCGGTGC TGCCTTATCT

A.tri_v2 CTACCAATCT CGGAATCGCC AATCCTGCAG GCCTCGGTGC TGCCTTATCT

A.tri_v3 CTACCAATCT CGGAATCGCC AATCCTGCAG GCCTCGGTGC TTCCTTATCT

A.tri_v4 CTACCAATCT CGGAATCGCC AATCCTGCAG GCCTCGGTGC TTCCTTATCT

A.aur_v1 CTAATAATCT GGGTATCGGC AACGCTGCAG GCCTCGGTAA TGCCTTATCT

A.aur_v2 CTAATAATCT CGGTATCGGC AACGCTGCAG GCCTCGGTAA TGCCTTATCT

A.aur_v3 CTAATAATCT CGGTATCGGC AACGCTGCAG GCCTCGGTAA TGCCTTATCT

A.aur_v4 CTAATAATCT CGGTATCGGC AACGCTGCAG GCCTCGGTAA TGCCTTATCT

**A.arg_TuSp1** CAAGCTGTCT CTTCGGTCGG CGTGGGAGCC AGTTCCGGCA CGTA 594

**A.arg_TuSp1ψ** CAAGCTGTCT CTTCGGTCGG CGTGGGAGCC AGTTCCGGCA CGTA

A.arg_a CAAGCTGTCT CTTCGGTCGG CGTGGGAGCC AGTTCCGGCA CGTA

A.arg_v2 CAAGCTGTCT CTTCGGTCGG CGTGGGAGCC AGT-CCGGCA CGTA

A.arg_v3 CAAGCTGTCT CTTCGGTCGG CGTGGGAGCC AGT-CCGGCA CGTA

A.arg_v4 CAAGCTGTCT CTTCGGTCGG CGTGGGAGCC AGT-CCGGCA CGTA

A.bru_a CAAGCTGTCT CTTCAGTAGG CGTAGGAGCC AGTTCCAGCA CGTA

A.bru_b* CAAGCTGTCT CTTCAGTCGG CGTTGGAGCC AGTTCCAGCA CGTA

A.tri_v1 CAAGCTGTCT CTTCAGCCGG CGTTGGAGCC AGTGCCGGCA CGTA

A.tri_v2 CAAGCTGTCT CTTCAACCGG CGTTGGAGCC AGTGCCGGCA CGTA

A.tri_v3 CAAGCTGTCT CTGCAGCCGG CGTTGGAGCC AGTGCAGGCA CGTA

A.tri_v4 CAAGCTGTCT CTGCAGCCGG CGTTGGAGCC AGTGCAGGCA CGTA

A.aur_v1 CAAGCTGTCT CTTCAGTCGG CGTTGGAGCC AGTTCCAGCA CGTA

A.aur_v2 CAAGCTGTCT CTTCAGTCGG CGTTGGAGCC AGTTCCAGCA CGTA

A.aur_v3 CAAGCTGTCT CTTCAGTCGG CGTTGGAGCC AGTTCCAGCA CGTA

A.aur_v4 CAAGCTGTCT CTTCAGTCGG CGTTGGAGCC AGTTCCAGCA CGTA

Figure S5. Nucleotide alignment of 3’ region PCR amplified variants (numbered) from *A. argentata, A. aurantia,* and *A. trifasciata* with *Argiope TuSp1* sequences (lettered). Alignment gaps indicated by dashes. GenBank accession numbers and full names for variants and downloaded sequences in Tables S2, S3.

**A.arg_TuSp1** CAATACCCTC TCAGCTCTCG GAAACGTTGC TTATCAACTA GGCTTCAACG 50

**A.arg_TuSp1ψ** CAATACCCTC TCAGCTCTCG GAAACGTTGC TTATCAACTA GGCTTCAACG

A.arg_v1 CAATACCCTC TCAGCTCTCG GAAACGTTGC TTATCAACTA GGCTTCAACG

A.arg_v2 CAATACCCTC TCAGCTCTCG GAAACGTTGC TTATCAACTA GGCTTCAACG

A.arg_v3 CAATACCCTC TCAGCTCTCG GAAACGTTGC TTATCAACTA GGCTTCAACG

A.arg_b CAATACCCTC TCAGCTCTCG GAAACGTTGC TTATCAACTA GGCTTCAACG

A.arg_c CAATACCCTC TCAGCTCTCG GAAACGTTGC TTATCAACTA GGCTTCAACG

A.tri_v1 CACTTCCCTC TCAGCTCTCG GCAACGTCGC TTATCAGTTG GGCTACAACG

A.tri_v2 CACTTCCCTC TCAGCTCTCG GCAAGGTCGC TTATCAGTTG GGCTACAACG

A.tri_v3 CACTTCCCTC TCGGCTCTCG GCAACGTCGC TTATCAGTTG GGCTTCAACA

A.tri_v4 CACTTCCCTC TCGGCTCTCG GCAACGTCGC TTATCAGTTG GGCTTCAACA

A.tri_v5 CACTTCCCTC TCGGCTCTCG GCAACGTCGC TTATCAGTTG GGCTTCAACA

A.tri_v6 CACTTCCCTC TCGGCTCTCG GCAACGTCGC TTATCAGTTG GGCTTCAACA

A.aur_a CAATTCCCTC TCAGCTCTCG GCAACGTAGC GTATCAATTA GGCTTCAACG

A.aur_b CAATTCNCTC TCAGCTCTCG GCAACGTAGS NTATCAATTA GGCTTCAACG

A.aur_v1 CAATTCTCTC TCAGCTCTCG GCAACGTTGC TTATCAATTA GGCTTCAACG

A.aur_v2 CAATTCACTC TCAGCTCTCG GCAACGTAGG TTATCAATTA GGCTTCAACG

A.aur_v3 CAATTCCCTC TCAGCTCTCG GCAACGTAGG TTATCAATTA GGCTTCAACG

A.bru_b CAATTCCCTC TCAGCTCTCG GCAACGTTGC TTATCAATTA GGCTTCAACG

A.bru_a CAATTCCCTC TCAGCTCTCG GCAACGTAGG TTATCAATTA GGCTTCAACG

A.gem CACTTCCCTC TCAGCTGTCG GAAACGTTGG TTATCAATTA GGCTTAAAGG

C.mol CACTTCCCTC TCAGCCCTCG GCAACGTTGC CTATCAATTA GGCTTCAACG

G.hep CACTTCCCTC TCCGCTCTCG GCAACGTTGC TTATCAATTG GGCTTCAACG

**A.arg_TuSp1** TAGCTAATAC TCTCGGTCTC GGCAACGCCG CAGGCCTCGG TGCCGCCTTA 100

**A.arg_TuSp1ψ** TAGCTAATAC TCTCGGTCTC GGCAACGCCG CAGGCCTCGG TGCCGCCTTA

A.arg_v1 TAGCTAATAC TCTCGGTCTC GGCAACGCCG CAGGCCTCGG TGCCGCCTTA

A.arg_v2 TAGCTAATAC TCTCGGTCTC GGCAACGCCG CAGGCCTCGG TGCCGCCTTA

A.arg_v3 TAGCTAATAC TCTCGGTCTC GGCAACGCCG CAGGCCTCGG TGCCGCCTTA

A.arg_b TAGCTAATAC TCTCGGTCTC GGCAACGCCG CAGGCCTCGG TGCCGCCTTA

A.arg_c TAGCTAATAC TCTCGGTCTC GGCAACGCCG CAGGCCTCGG TGCCGCCTTA

A.tri_v1 CAGCTACCAA TCTTGGAATC GCCAATCCTG CAGGCCTCGG TGCTTCCTTA

A.tri_v2 CAGCTACCAA TCTCGGAATC GCCAATCCTG CAGGCCTCGG TGCTTCCTTA

A.tri_v3 CAGCTACCAA TCTCGGAATC GCCAATCCTG CAGGCCTCGG TGCTGCCTTA

A.tri_v4 CAGCTACCAA TCTCGGAATC GCCAATCCTG CAGGCCTCGG TGCTGCCTTA

A.tri_v5 CAGCTACCAA TCTCGGAATC GCCAATCCTG CAGGCCTCGG TGCTGCCTTA

A.tri_v6 CAGCTACCAA TCTCGGAATC GCCAATCCTG CAGGCCTCGG TGCTGCCTTA

A.aur_a TAGCTAATAC TCTCGGTATC GGCAACGCTG CAGGCCTCGG TAATGCCTTA

A.aur_b TAGCTAATAM TCTGGGTATC GGCAACGCTG CAGGCCTCGG TAATGCCTTA

A.aur_v1 TAGCTAATAA TCTCGGTATC GGCAACGCTG CAGGCCTCGG TAATGCCTTA

A.aur_v2 TAGCTAATAA TCTGGGTATC GGCAACGCTG CAGGCCTCGG TAATGCCTTA

A.aur_v3 TAGCTAATAC TCTGGGTATC GGCAACGCTG CAGGCCTCGG TAATGCCTTA

A.bru_b TAGCTAACAC TCTCGGTATC GGCAACGCTC CAGGCCTCGG TGCTGCCTTA

A.bru_a TAGCTAATAC TCTCGGTATC GGAAACGCTC CAGGCCTCGG TAATGCCTTA

A.gem TGGCTAATTC TCTCGGACTC GGCAACGCTC AAGCC----- ----------

C.mol TAGGTACTTC TCTCGGACTT ---AACGCTC AAGCACTCGG TGCTGCCTTA

G.hep TAGCTAACAA TCTCGGACTC GGCAACGCTG CAGGCCTCGG AGCTGCCCTT

**A.arg_TuSp1** TCTCAAGCTG TCTCTTCGGT CGGCGTGGGA GCCAGTTCCG CCACGTACGC 150

**A.arg_TuSp1ψ** TCTCAAGCTG TCTCTTCGGT CGGCGTGGGA GCCAGTTCCG GCACGTACGC

A.arg_v1 TCTCAAGCTG TCTCTTCGGT CGGCGTGGGA GCCAGTTCCG CCACGTACGC

A.arg_v2 TCTCAAGCTG TCTCTTCGGT CGGCGTGGGA GCCAGTTCCG GCACGTACGC

A.arg_v3 TCTCAAGCTG TCTCTTCGGT CGGCGTGGGA GCCAGTTCCG GCACGTACGC

A.arg_b TCTCAAGCTG TCTCTTCGGT CGGCGTGGGA GCCAGTTCCG CCACGTACGC

A.arg_c TCTCAAGCTG TCTCTTCGGT CGGCGTGGGA GCCAGTTCCG CCACGTACGC

A.tri_v1 TCTCAAGCTG TCACTGCAGC CGGCGTTGGA GCCAGTGCAG GCACGTACGC

A.tri_v2 TCTCAAGCTG TCTCTGCAGC CGGCGTTGGA GCCAGTGCAG GCACGTACGC

A.tri_v3 TCTCAAGCTG TCTCTTCAGC CGGCGTTGGA GCCAGTGCCG GCACGTACGC

A.tri_v4 TCTCAAGCTG TCTCTTCAGC CGGCGTTGGA GCCAGTGCCG GCACGTACGC

A.tri_v5 TCTCAAGCTG TCTCTTCAGC CGGCGTTGGA GCCAGTGCCG GCACGTACGC

A.tri_v6 TCTCAAGCTG TCTCTTCAGC CGGCGTTGGA GCCAGTGCCG GCACGTACGC

A.aur_a TCTCAAGCTG TCTCTTCAGT CGGCGTTGGA GCCAGTTCCA GCACGTACGC

A.aur_b TCTCAAGCTG TCTCTTCAGT CGGCGTTGGA GCCAGTTCCA GCACGTACGC

A.aur_v1 TCTCAAGCTG TCTCTTCAGT CGGCGTTGGA GCCAGTTCCA GCACGTACGC

A.aur_v2 TCTCAAGCTG TCTCTTCAGT CGGCGTTGGA GCCAGTTCCA GCACGTACGC

A.aur_v3 TCTCAAGCTG TCTCTTCAGT CGGCGTTGGA GCCAGTTCCA GCACGTACGC

A.bru_b TCTCAAGCTG TCTCTTCAGT CGGCGTTGGA GCTAGTTCCA GCACGTACGC

A.bru_a TCGCAAGCTG TGTCTTCAGT CGGCGTTGGA GCCAGTTCCA GCGCGTACGC

A.gem ---------- ---------- ---------- ---------- ----------

C.mol TCTCAAGCTG TCTCTGCTGT GGGCGTTGGA GCCAGTTCAA GCGCATACGC

G.hep TCTCAAGCTG TCTCTTCAGT CGGCGTTGGA GCCAGTTCCT TCGCTTACGC

**A.arg_TuSp1** TAATGCCGTT TCCAATGCAG TTGGACAATT CTTAGCCGGT CAAGGTATTT 200

**A.arg_TuSp1ψ** TAATGCCGTT TCCAATGCAG TTGGACAATT CTTAGCCGGT CAAGGTATTT

A.arg_v1 TAATGCCGTT TCCAATGCAG TTGGACAATT CTTAGCCGGT CAAGGTATTT

A.arg_v2 TAATGCCGTT TCCAATGCAG TTGGACAATT CTTAGCCGGT CAAGGTATTT

A.arg_v3 TAATGCCGTT TCCAATGCAG TTGGACAATT CTTAGCCGGT CAAGGTATTT

A.arg_b TAATGCCGTT TCCAATGCAG TTGGACAATT CTTAGCCGGT CAAGGTATTT

A.arg_c TAATGCCGTT TCCAATGCAG TTGGACAATT CTTAGCCGGT CAAGGTATTT

A.tri_v1 TAATATCATT TCCAATGTAG TTGGACAATA CTTAGCCTCT CAGGGTGTTT

A.tri_v2 TAATATCGTT TCCAATGTAG TTGGACAATA CTTAGCCTCT CAGGGTGTTT

A.tri_v3 TAATATCATT TCCAATGTAG TTGGACAATA CTTAGCCTCT CAGGGTGTTT

A.tri_v4 TAATATCATT TCCAATGCAG TTGGACAATA CTTAGCCGCT CAGGGTATTT

A.tri_v5 TAATATCGTT TCCAATGCAG TTGGACAATA CTTAGCCGCT CAGGGTGTTT

A.tri_v6 TAATATCATT TCCAATGCAG TTGGACAATA CTTAGCCGCT CAGGGTATTT

A.aur_a TAATGCCGTT TCCAATGCAG TGGGACAATT TTTAGCTGGT CAGGGCGTTT

A.aur_b TAATGCCGTT TCCAATGCAG TTGGACAATT TTTAGCTGGT CAGGGCGTTT

A.aur_v1 TAATGCCGTT TCCAATGCAG TTGGACAATT CTTAGCCACT CAGGGCGTTT

A.aur_v2 TAATGCCGTT TCCAATGCAG TTGGACAATT TTTAGCTGGT CAGGGCGTTT

A.aur_v3 TAATGCCGTT TCCAATGCAG TTGGACAATT TTTAGCTGGT CAGGGCGTTT

A.bru_b TAATGTCGTT TCCAATGCAG TTGGACAATT CTTAGCCGGT CAGGGCGTTT

A.bru_a TAATGCCGTT TCCAATGCAG TTGGGCAATT CTTAGCCGGC CAGGGCGTTC

A.gem ---------- ---------- ---------- -TTAGCCAGT CAGGGTATTT

C.mol GAATGCCGTT TCCAATGCAA CTGGACAATT CTTAGCCGGC CAAGGTGTTT

G.hep TAATGCCGTC TCCAATGCAG TTGCACAATT CTTATCGGCT CAAGGTATTT

**A.arg_TuSp1** TGAATGCGGC CAATGCAGCT TCCCTTGCCA CCTCCTTTGC TAATGCTGTA 250

**A.arg_TuSp1ψ** TGAACGCGGC CAATGCAGCT TCCCTTGCCA CCTCCTTTCC TAATGCTGTA

A.arg_v1 TGAATGCGGC CAATGCAGCT TCCCTTGCTA CCTCCTTTGC TAATGCTGTA

A.arg_v2 TGAACGCGGC CAATGCAGCT TCCCTTGCCA CCTCCTTTCC TAATGCTGTA

A.arg_v3 TGAACGCGGC CAATGCAGCT TCCCTTGCCA CCTCCTTTCC TAATGCTGTA

A.arg_b TGAATGCGGC CAATGCAGCT TCCCTTGCCA CCTCCTTTGC TAATGCTGTA

A.arg_c TGAATGCGGC CAATGCAGCT TCCCTTGCCA CCTCCTTTGC TAATGCTGTA

A.tri_v1 TGAACTCGGC CAATGCAGGT TCCCTTGCCT CCTCCTTCGC CAGTGCTCTC

A.tri_v2 TGAACTCGGC CAATGCAGGT TCCCTTGCCT CCTCCTTCGC CAGTGCTCTC

A.tri_v3 TGAACTCGGC CAATGCAGGT TCCCTTGCCT CCTCCTTCGC CAGTGCTCTC

A.tri_v4 TGAACGCGGC CAATGCAGGT TCCCTTGCCT CCACCTTCGC CAGTGCTCTC

A.tri_v5 TGAACGCGGC CAATGCAGGT TCCCTTGCCT CCACCTTCGC CAGTGCTCTC

A.tri_v6 TGAACGCGGC CAATGCAGGT TCCCTTGCCT CCACCTTCGC CAGTGCTCTC

A.aur_a TGAACGCTGG CAATGCAGGT TCTCTTGCCT CCTCGTTTGC CAATGCTCTA

A.aur_b TGAACGCTGG CAATGCAGGT TCTCTTGCCT CCTCGTTTGC CAATGCTCTA

A.aur_v1 TGAACGCTGG CAATGCAGGT TCCCTTGCCT CCTCCTTCGC CAATGCTCTA

A.aur_v2 TGAACGCTGG CAATGCAGGT TCTCTTGCCT CCTCGTTTGC CAATGCTCTA

A.aur_v3 TGAACGCTGG CAATGCAGGT TCTCTTGCCT CCTCGTTTGC CAATGCTCTA

A.bru_b TGAACGCGGC CAATGCAGGT TCCCTTGCCT CCTCCTTTGC CAGTGCTCTA

A.bru_a TGAACGCTGG CAATGCAGGT TCCCTTGCCT CCTCCTTTGC CAATGCTCTG

A.gem TGAACGCGGC CAATGCAGGT TCCCTAGCCT CCTCCTTTGC CAGTGCTCTA

C.mol TGAACGCGAG CAACGCAGCT GCCCTTGCCT CCTCGTTTGC CAGTGCTCTA

G.hep TGAACGCTGC CAATGCAGGT TCCCTTGCTT CTTCCTTTGC CAGTGCTCTC

**A.arg_TuSp1** TCT------- ---------- ----TCATCT GCTCTTGCTG CTGTAAGTCG 300

**A.arg_TuSp1ψ** TCT------- ---------- ----TCATCT GCTCTTGCTG CTNTAAGTCG

A.arg_v1 TCT------- ---------- ----TCATCT GCTCTTGCTG CTGTAAGTCG

A.arg_v2 TCT------- ---------- ----TCATCT GCTCTTGCTG CTNTAAGTCG

A.arg_v3 TCT------- ---------- ----TCATCT GCTCTTGCTG CTNTAAGTCG

A.arg_b TCT------- ---------- ----TCATCT GCTCTTGCTG CTGTAAGTCG

A.arg_c TCT------- ---------- ----TCATCT GCTCTTGCTG CTGTAAGTCG

A.tri_v1 TCA------- ---------- ----GGATCC GCTCTTTCAA TTGGTAGTCG

A.tri_v2 TCA------- ---------- ----GGATCC GCTCTTTCAA TTGGTAGTCG

A.tri_v3 TCA------- ---------- ----GGATCC GCTCTTTCAA TTGGTAGTCG

A.tri_v4 TCA------- ---------- ----GCATCC GCTCTTTCAA CTGGAAGTCG

A.tri_v5 TCA------- ---------- ----GCATCC GCTCTTTCAA CTGGAAGTCG

A.tri_v6 TCA------- ---------- ----GCATCC GCTCTTTCAA CTGGAAGTCG

A.aur_a TCA------- ---------- ----AATTCC GCTCTTTCAG TTGGCAGTCG

A.aur_b TCA------- ---------- ----AATTCC GCTCTTTCAG TTGGCAGTCG

A.aur_v1 TCA------- ---------- ----AATTCC GCTCTTTCAG TTGGCAGTCG

A.aur_v2 TCA------- ---------- ----AATTCC GCTCTTTCAG TTGGCAGTCG

A.aur_v3 TCA------- ---------- ----AATTCC GCTCTTTCAG TTGGCAGTCG

A.bru_b TCA------- ---------- ----AATTCC GCTCTTTCAA TTGGTAGTCG

A.bru_a TCA------- ---------- ----AATTCC GCTCTTTCAG TTGGCAGTCG

A.gem TCA------- ---------- ----GCATCT GCTGGTTCGG TTGGAAACCG

C.mol TCA------- ---------- ----ACATCA GCTAGTTCAA TTACAGGACG

G.hep TCATCCGTCG CGGCCTCTGC ATCTTCTTCC GCTCTTTCAC TTGGAAGTAC

**A.arg_TuSp1** CATCAGTTCT ---CCTTCTT ATGGCGCTTT TGCCTCTGTT ---------C 350

**A.arg_TuSp1ψ** CGTCAGTTCT ---CCTTCTT ATGGCGCTTT TGCTTCTGTT ---------C

A.arg_v1 CATCAGTTCT ---CCTTCTT ATGGCGCTTT TGCCTCTGTT ---------C

A.arg_v2 CGTCAGTTCT ---CCTTCTT ATGGCGCTTT TGCTTCTGTT ---------C

A.arg_v3 CGTCAGTTCT ---CCTTCTT ATGGCGCTTT TGCTTCTGTT ---------C

A.arg_b CATCAGTTCT ---CCTTCTT ATGGCGCTTT TGCCTCTGTT ---------C

A.arg_c CATCAGTTCT ---CCTTCTT ATGGCGCTTT TGCCTCTGTT ---------C

A.tri_v1 TGGCAGCACT ---CCTTCTT ATGGGGCTCT AAGTCCTATT ----------

A.tri_v2 TGGCAGCACT ---CCTTCTT ATGGGGCTCT AAGTCCTATT ----------

A.tri_v3 TGGCAGCACT ---CCTTCTT ATGGGGCTCT AAGTCCTATT ----------

A.tri_v4 TGGCAGCACT ---CCTTCTT ATGGTGCTCT AAGTCCTATT ----------

A.tri_v5 TGGCAGCACT ---CCTTCTT ATGGTGCTCT AAGTCCTATT ----------

A.tri_v6 TGGCAGCACT ---CCTTCTT ATGGTGCTCT AAGTCCTATT ----------

A.aur_a CGTCAGTTCT ---CCTTCTT ATGGCGCCTT AAGTCCTATT GCAGCTGGTC

A.aur_b CRTCAGTTCT ---CCTTCTT ATGGCGCCTT AAGTCCTATT GCAGCTGGTC

A.aur_v1 TGTCAGTTCT ---ACTTCTT ATGGCGCCTT AAGTCCTATT GCAGCTGGTC

A.aur_v2 CGTCAGTTCT ---CCTTCTT ATGGCGCCTT AAGTCCTATT GCAGCTGGTC

A.aur_v3 CATCAGTTCT ---CCTTCTT ATGGCGCCTT AAGTCCTATT GCAGCTGGTC

A.bru_b CGTCAGTTCT ---CCTTCTT ATGGCGTCTT TAGTCCTATT GCAGCTGGTC

A.bru_a CGTCAGTTCT ---CCTTCTT ATGGCGCCTT AAGTCCTATT GCAGCTGGTC

A.gem TAGCAGTGCA GGACCTTCTG CTGTCGGCTT GGGTGGTGTT TCAGCTGTTC

C.mol GGTTGGTGCC AGTCCTTACT CTGGTGCA-- -AGTCCAGTT TCAGCTACTC

G.hep CTTCCCTGCT ---CCATCTT ACGGA----- ---------- ----------

**A.arg_TuSp1** CCAAATTTGT TCCCTCTAAC CTGAATGCTG GAGGTGTTTC GTTCGGCGAA 400

**A.arg_TuSp1ψ** CCAAATTTAT TCCCGCTGAC CTGGATGCTG GAGGCATTTC GATCGGCAAA

A.arg_v1 CCAAATTTGT TCCCTCTAAC CTGAATGCTG GAGGTGTTTC GTTCGGCGAA

A.arg_v2 CCAAATTTAT TCCCGCTGAC CTGGATGCTG GAGGCATTTC GATCGGCAAA

A.arg_v3 CCAAATTTAT TCCCGCTGAC CTGGATGCTG GAGGCATTTC GATCGGCAAA

A.arg_b CCAAATTTGT TCCCTCTAAC CTGAATGCTG GAGGTGTTTC GTTCGGCGAA

A.arg_c CCAAATTTGT TCCCTCTAAC CTGAATGCTG GAGGTGTTTC GTTCGGCGAA

A.tri_v1 ---------- -TCCACTGGT CTGAATGCTG GAGGCGTTTT GGTTGGCGGA

A.tri_v2 ---------- -TCCACTGGT CTGAATGCTG GAGGCGTTTT GGTTGGCGGA

A.tri_v3 ---------- -TCCACTGGT CTGAATGCTG GAGGCGTTTT GGTTGGCGGA

A.tri_v4 ---------- -TCCACTGGT CTGAATGCTG GAGGCGTTTC GGTTGGCGGA

A.tri_v5 ---------- -TCCACTGGT CTGAATGCTG GAGGCGTTTC GGTTGGCGGA

A.tri_v6 ---------- -TCCACTGGT CTGAATGCTG GAGGCGTTTC GGTTGGCGGA

A.aur_a CCAATTTTAT TTCCACTGGC CTTAAT---- ---------- -GTCGGCGGG

A.aur_b CCAATTTTAT TTCCACTGGC CTTAAT---- ---------- -GTCGGCGGG

A.aur_v1 CCAATTTTAT TTCCACAGGC CTTAATGTCG GTGGTGCTTC GGTCGGCGGG

A.aur_v2 CCAATTTTAT TTCCACTGGC CTTAAT---- ---------- -GTCGGCGGG

A.aur_v3 CCAATTTTAT TTCCACTGGC CTTAAT---- ---------- -GTCGGCGGG

A.bru_b CCAATTCTAT TTCCACTGGC CTTAATGTCG GTGGTGCTTC GATCGGCGGG

A.bru_a CCAATTTTAT TTCCACTGGC CTTAATGTCG GTGGGGCTTC GGTCGGCGGG

A.gem CCGGCTTTAT TTCTGCTACC CCAGTAGTAG GTGGG----- ----------

C.mol CTGGCCTTAT TTCTGCTGTT CCTCAGGGTG TAGGGTTTTC GTTCGGTCGA

G.hep ---------- ---------- TTTAATAATG GAGGTGTTTC AGTCGGTGGG

**A.arg_TuSp1** CCTTTTGCTG CTCTTAGTCA ATCCGTGCCC ACTGACCTTC AATCTGCTTT 450

**A.arg_TuSp1ψ** CCTTATGCTG CTCTCAGTCA ATTTTTGCCT ACGAACATTC AAACTGCTTT

A.arg_v1 CCTTTTGCTG CTCTTAGTCA ATCCGTGCCC ACTGACCTTC AATCTGCTTT

A.arg_v2 CCTTATGCTG CTCTCAGTCA ATTTTTGCCT ACGAACATTC AAACTGCTTT

A.arg_v3 CCTTATGCTG CTCTCAGTCA ATTTTTGCCT ACGAACATTC AAACTGCTTT

A.arg_b CCTTTTGCTG CTCTTAGTCA ATCCGTGCCC ACTGACCTTC AATCTGCTTT

A.arg_c CCTTTTGCTG CTCTTAGTCA ATCCGTGCCC ACTGACCTTC AATCTGCTTT

A.tri_v1 CCTTCCGCCT TCCTTAGTCA GGCATTGCCC GTTGGTCTTC AAACTGCTTT

A.tri_v2 CCTTCCGCCT TCCTTAGTCA GGCATTGCCC GTTGGTCTTC AAACTGCTTT

A.tri_v3 CCTTCCGCCT TCCTTAGTCA GGCATTGCCC GTTGGTCTTC AAACTGCTTT

A.tri_v4 CCTTCCGCCT TCATTAGTCA GGCATTGCCC GTTGGTCTTC AAACTGCTTT

A.tri_v5 CCTTCGGCCT TCATTAGTCA GGCATTGCCC GTTGGTCTTC AAACTGCTTT

A.tri_v6 CCTTCCGCCT TCATTAGTCA GGCATTGCCC GTTGGTCTTC AAACTGCTTT

A.aur_a CCTTTTACCA CCCTTAGCCA GTCGTTGCCC ACGAGCCTTC AAACTGCTTT

A.aur_b CCTTTTACCA CCCTTAGCCA GTCGTTGCCC ACGAGCCTTC AAACTGCTTT

A.aur_v1 CCTTTTGCCA CCCTTAGTCA ATCGTTGCCC ACGAGCCTAC AAACTGCTTT

A.aur_v2 CCTTTTACCA CCCTTAGCCA GTCGTTGCCC ACGAGCCTTC AAACTGCTTT

A.aur_v3 CCTTTTACCA CCCTTAGCCA GTCGTTGCCC ACGAGCCTTC AAACTGCTTT

A.bru_b CCTTTTGCCA CCCTTAGTCA ATCGTTGCCC ACGAGCCTTC AAACTGCTTT

A.bru_a CCTTTTGACT CCCTTAGTCA ATCGTTGCCC ACGAGCCTTC AAACTGCTTT

A.gem CCAGTTACTG TCAATGGCCA AGTTTTGCCC GCCGCTCTTC AAACTGCTTT

C.mol TCGGAC---- --------CA ATTGCTGCCA TCTTCTCTTC AAAGTGCTTT

G.hep CCTTTTGCCA CAGTTGGTCA ATCGTTGCCT ATTAGCCTCC AAACTGCATT

**A.arg_TuSp1** AGCTCCTATT GCTTCGTCTT CTGGCTTAGG TTCCTCTGCT GCCTCTGCCA 500

**A.arg_TuSp1ψ** AGCTCCTATT GCGTCTTCTT CAGGCTTAGG TTCCTCTGCT GCCTCTGACA

A.arg_v1 AGCTCCTATT GCTTCGTCTT CTGGCTTAGG TTCCTCTGCT GCCTCTGCCA

A.arg_v2 AGCTCCTATT GCGTCTTCTT CAGGCTTAGG TTCCTCTGCT GCCTCTGACA

A.arg_v3 AGCTCCTATT GCGTCTTCTT CAGGCTTAGG TTCTTCTGCT GCCTCTGACA

A.arg_b AGCTCCTATT GCTTCGTCTT CTGGCTTAGG TTCCTCTGCT GCCTCTGCCA

A.arg_c AGCTCCTATT GCTTCGTCTT CTGGCTTAGG TTCCTCTGCT GCCTCTGCCA

A.tri_v1 AGCTCCTATT GTTTCTTCTT CTGGCTTGGG CTCATCTTCT GCTGCAGCCA

A.tri_v2 AGCTCCTATT GTTTCTTCTT CTGGCTTGGG CTCATCTTCT GCTGCAGCCA

A.tri_v3 AGCTCCTATT GTTTCTTCTT CTGGCTTGGG CTCATCTTCT GCTGCAGCCA

A.tri_v4 AGCTCCTATT GTTTCTTCTT CTGGCTTGGG CTCATCTTCT GCTGCAGCCA

A.tri_v5 AGCTCCTATT GTTTCTTCTT CTGGCTTGGG CTCATCTTCT GCTGCAGCCA

A.tri_v6 AGCTCCTATT GTTTCTTCTT CTGGCTTGGG CTCATCTTCT GCTGCAGCCA

A.aur_a AGCTCCTATT GTTTCTTCTT CAGGCTTAGG CTCATCCGCT GCCACTGCCA

A.aur_b AGCTCCTATT GTTTCTTCTT CAGGCTTAGG CTCATCCGCT GCCACTGCCA

A.aur_v1 AGCTCCTATT GTTTCTTCTT CAGGCTTAGG CTCATCCGCT GCCACTGCCA

A.aur_v2 AGCTCCTATT GTTTCTTCTT CAGGCTTAGG CTCATCCGCT GCCACTGCCA

A.aur_v3 AGCTCCTATT GTTTCTTCTT CAGGCTTAGG CTCATCCGCT GCCACTGCCA

A.bru_b AGCTCCTATT GTTTCTTCTT CAGGCTTAGG TTCATCTGCT GCCACTGCCA

A.bru_a AGCTCCTATT GTTTCTTCTT CAGGCTTAGG CTCATCTGCT GCCACTGCCA

A.gem AGCTCCAGTT GTTACTTCTT CTGGTTTGGC CTCATCTGCT GCCAGTGCCA

C.mol AGCTCCAATT ATTTCTTCTT CAGGCTTAGC ATCACCTGAT GCCACTGCCA

G.hep AGCTCCTGTT CTTTCTTCCA CAGGCTTAGG TTCATCTGCT GCCTCAGCCA

**A.arg_TuSp1** GAGTGAGTAG TTTAGCCAAT TCCGTTGCTT CTGCTATTTC TTCATCTGGA 550

**A.arg_TuSp1ψ** GAGTGAGTAG TTTAGCCAAT TCCCTTGCTT CCGCAATTTC TTCATCTGGA

A.arg_v1 GAGTGAGTAG TTTAGCCAAT TCCGTTGCTT CTGCTATTTC TTCATCTGGA

A.arg_v2 GAGTGAGTAG TTTAGCCAAT TCCCTTGCTT CCGCAATTTC TTCATCTGGA

A.arg_v3 GAGTGAGTAG TTTAGCCAAT TCCCTTGCTT CCGCAATTTC TTCATCTGGA

A.arg_b GAGTGAGTAG TTTAGCCAAT TCCGTTGCTT CTGCTATTTC TTCATCTGGA

A.arg_c GAGTGAGTAG TTTAGCCAAT TCCGTTGCTT CTGCTATTTC TTCATCTGGA

A.tri_v1 GGGTCAGTAG TTTAGCCAAT TCCTTTGCTT CAGCTATCTC TTCATCGGGT

A.tri_v2 GGGTCAGTAG TTTAGCCAAT TCCTTTGCTT CAGCTATCTC TTCATCGGGT

A.tri_v3 GGGTCAGTAG TTTAGCCAAT TCCTTTGCTT CAGCTATCTC TTCATCGGGT

A.tri_v4 GGGTCAGTAG TTTAGCCAAT TCCTTTGCTT CAGCTATCTC TTCATCTGGC

A.tri_v5 GGGTCAGTAG TTTAGCCAAT TCCTTTGCTT CAGCTATCTC TTCATCTGGT

A.tri_v6 GGGTCAGTAG TTTAGCCAAT TCCTTTGCTT CAGCTATCTC TTCATCGGGT

A.aur_a GGGTGAGGAG TTTAGCCAAC TCCATTGCTT CTGCGATTTC TTCATCTGGA

A.aur_b GGGTGAGGAG TTTAGCCAAC TCCATTGCTT CTGCGATTTC TTCATCTGGA

A.aur_v1 GGGTGAGTAG TTTAGCCAAC TCCATTGCTT CTGCGATTTC TTCATCTGGA

A.aur_v2 GGGTGAGGAG TTTAGCCAAC TCCATTGCTT CTGCGATTTC TTCATCTGGA

A.aur_v3 GGGTGAGGAG TTTAGCCAAC TCCATTGCTT CTGCGATTTC TTCATCTGGA

A.bru_b GGGTGAGTAG TTTAGCCAAC TCCATTGCTT CTGCGATTTC TTCATCTGGA

A.bru_a GGGTGAGTAG TTTAGCCAAC TCCTTTGCTT CTGCGATTTC TTCATCTGGA

A.gem GAGTGAGTAG CTTAGCCCAA TCAATTGCTT CTGCGATTTC TTCATCCGGT

C.mol GAGTCAGTAG TTTATCTAAT TCCATAGCTT CAGCAATTTC TTCATCCGGA

G.hep GGGTTGGTAG TTTAGCCAGC TCCATTGCTT CAGCTATTTC TTCATCCGGA

**A.arg_TuSp1** GGCTCCCTCA GTGTTCCAAC CTTCTTGAAT TTTCTTTCAT CCGTTGGGGC 600

**A.arg_TuSp1ψ** GGCTCCCTCA GCGTTCGAAC CTTTTTGAAT TTTCTTTCAA ACGTTGGGGC

A.arg_v1 GGCTCCCTCA GTGTTCCAAC CTTCTTGAAT TTTCTTTCAT CCGTTGGGGC

A.arg_v2 GGCTCCCTCA GCGTTCGAAC CTTTTTGAAT TTTCTTTCAA ACGTTGGGGC

A.arg_v3 GGCTCCCTCA GCGTTCGAAC CTTTTTGAAT TTTCTTTCAA ACGTTGGGGC

A.arg_b GGCTCCCTCA GTGTTCCAAC CTTCTTGAAT TTTCTTTCAT CCGTTGGGGC

A.arg_c GGCTCCCTCA GTGTTCCAAC CTTCTTGAAT TTTCTTTCAT CCGTTGGGGC

A.tri_v1 GGTTCCCTCA GTGTTCCAAC CTTCTTGAGT CTTCTTTCCT CCGTTGGGGC

A.tri_v2 GGTTCCCTCA GTGTTCCAAC CTTCTTGAGT CTTCTTTCCT CCGTTGGGGC

A.tri_v3 GGTTCCCTCA GTGTTCCAAC CTTCTTGAGT CTTCTTTCCT CCGTTGGGGC

A.tri_v4 GGTTACCTCA GTGTTCCAAC CTTCTTGAGT CTTCTTTCCT CCGTTGGGGC

A.tri_v5 GGTTACCTCA GTGTTCCGAC CTTCTTGAGT CTTCTTTCCT CCGTTGGGGC

A.tri_v6 GGTTCCCTCA GTGTTCCAAC CTTCTTGAGT CTTCTTTCCT CCGTTGGGGC

A.aur_a GGTTCCCTCA GTGTTCCAGC CTTCTTGAAT CTCCTCTCAT CCGTCGGGGC

A.aur_b GGTTCCCTCA GTGTTCCAGC CTTCTTGAAT CTCCTCTCAT CCGTCGGGGC

A.aur_v1 GGTTCCCTCA GTGTTCCAGC CTTCTTGAAT CTCCTCTCAT CCGTCGGGGC

A.aur_v2 GGTTCCCTCA GTGTTCCAGC CTTCTTGAAT CTCCTCTCAT CCGTCGGGGC

A.aur_v3 GGTTCCCTCA GTGTTCCAGC CTTCTTGAAT CTCCTCTCAT CCGTCGGGGC

A.bru_b GGTTCCCTCA GTGTTCCAAC CTTCTTGAAT CTTCTTTCAT CCATTGGGGC

A.bru_a GGTTCCCTCA GTGTTCCAAC CTTCTTGAAT CTTCTTTCAT CCGTTGGGGC

A.gem GGTACCTTGA GTGTTCCTAT CTTCTTGAAT CTTCTCTCAT CCGCTGGAGC

C.mol GGTTCTTTAA ATGTTCCTAC TTTTTTGAAT CTGCTCTCAT CAGTTGGATC

G.hep GGTTTCCTCA GTGTTCCAAC CTTCTTAACG CTTCTCTCAT CAGTTGGGTC

**A.arg_TuSp1** TCAAGTTAGC AGTAGTAGTT CTTTGAATTC CTCC---GAA GTTACAAACG 650

**A.arg_TuSp1ψ** TCAAGTTAGC AGTAGTAGTT CTTTGAGTTC CTCG---GCA GTTACAACCC

A.arg_v1 TCAAGTTAGC AGTAGTAGTT CTTTGAATTC CTCC---GAA GTTACAAACG

A.arg_v2 TCAAGTTAGC AGTAGTAGTT CTTTGAGTTC CTCG---GCA GTTACAACCC

A.arg_v3 TCAAGTTAGC AGTAGTAGTT CTTTGAGTTC CTCG---GCA GTTACAACCC

A.arg_b TCAAGTTAGC AGTAGTAGTT CTTTGAATTC CTCC---GAA GTTACAAACG

A.arg_c TCAAGTTAGC AGTAGTAGTT CTTTGAATTC CTCC---GAA GTTACAAACG

A.tri_v1 CCAAGTTAGT AGCAGCAGCT CTTTGAGTTC CTCG---CAA GTTACGAACG

A.tri_v2 CCAAGTTAGT AGCAGCAGCT CTTTGAGTTC CTCG---CAA GTTACGAACG

A.tri_v3 CCAAGTTAGT AGCAGCAGCT CTTTGAGTTC CTCG---CAA GTTACGAACG

A.tri_v4 CCAAGTTAGT AGCAGCAACT CTTTGAGTTC CTCG---CAA GTTACGAACG

A.tri_v5 CCAAGTTAGT AGCAGCAACT CTTTGAGTTC CTCGC---AA GTTACGAACG

A.tri_v6 CCAAGTTAGT AGCAGCAGCT CTTTGAGTTC CTCG---CAA GTTACGAACG

A.aur_a TCAAGTTAGT AGTAGCAGCT CTTTGAATTC CTCG---GAA GTTACAAATG

A.aur_b TCAAGTTAGT AGTAGCAGCT CTTTGAATTC CTCG---GAA GTTACAAATG

A.aur_v1 TCAAGTTAGT AGTAGCAGCT CTTTGAGTTC TTCCTCGGAA GTTACAACCC

A.aur_v2 TCAAGTTAGT AGTAGCAGCT CTTTGAATTC CTCG---GAA GTTACAAATG

A.aur_v3 TCAAGTTAGT AGTAGCAGCT CTTTGAATTC CTCG---GAA GTTACAAATG

A.bru_b GCAAGTTAGT AGTAGCAGTT CTTTGAGTTC TTCCTCGGAA GTTACAACCC

A.bru_a CCAAGTTAGT AGTAGCAGTT CTTTGAGTTC CTTG---GAA GTTACAAACG

A.gem ACAAGCTACT GCTAGCAGTT CTTTGAGTTC CTCG---CAA GTTACTAGCC

C.mol CCAAATTAGT GGCAGTAGTT CCCTGAGTTC TTCT---CAG ATAAGGAGCC

G.hep CCAAGTCGCT AGCAGCAGCT CTTTGAGTTC CTCT---GAA GTAACAAACG

**A.arg_TuSp1** AAGTGTTACT TGAAGCTATC GCGGCA 676

**A.arg_TuSp1ψ** AAGTTTTACT TGAAGCCATT GCGGCT

A.arg_v1 AAGTGTTACT TGAAGCTATC GCGGCA

A.arg_v2 AAGTTTTACT TGAAGCCATT GCGGCT

A.arg_v3 AAGTTTTACT TGAAGCCATT GCGGCT

A.arg_b AAGTGTTACT TGAAGCTATC GCGGCA

A.arg_c AAGTGTTACT TGAAGCTATC GCGGCA

A.tri_v1 AAGTCTTACT TGAGGCAATT GCTGCG

A.tri_v2 AAGTCTTACT TGAGGCAATT GCTGCG

A.tri_v3 AAGTCTTACT TGAGGCAATT GCTGCG

A.tri_v4 AAGTCTTACT TGAGGCAATT GCTGCG

A.tri_v5 AAGTCTTACT TGAGGCAATT GCTGCG

A.tri_v6 AAGTCTTACT TGAGGCAATT GCTGCG

A.aur_a AAGTTTTACT TGAAGCTATA GCGGCG

A.aur_b AAGTTTTACT TGAAGCTATA GCGGCG

A.aur_v1 AAGTTTTACT TGAAGCTATT GCGGCG

A.aur_v2 AAGTTTTACT TGAAGCTATA GCGGCG

A.aur_v3 AAGTTTTACT TGAAGCTATA GCGGCG

A.bru_b AAGTTTTACT TGAAGCTATT GCGGCG

A.bru_a AAGTTTTACT TGAAGCTATT GCGGCT

A.gem AAGTTTTGCT GGAAGGTATT GCAGCT

C.mol AAATTTTATT GGAGGGTATT GCTGCC

G.hep AAGTTTTACT TGAAACTATT TCGGCG
